# Phylogenetic and Functional Characterization of Culturable Endophytic Actinobacteria Associated With *Camellia* spp. for Growth Promotion in Commercial Tea Cultivars

**DOI:** 10.3389/fmicb.2020.00318

**Published:** 2020-02-28

**Authors:** Atlanta Borah, Debajit Thakur

**Affiliations:** Microbial Biotechnology Laboratory, Life Sciences Division, Institute of Advanced Study in Science and Technology, Guwahati, India

**Keywords:** 16S rRNA, antagonistic, *Camellia* spp., endophytic actinobacteria, tea growth promotion

## Abstract

Plant associated endophytic actinobacteria may contribute to plant growth and defense by direct or indirect methods. Our aim was to evaluate the plant growth promoting and antifungal activities of endophytic actinobacteria associated with *Camellia* spp. and related genera, *Eurya* to find potent plant growth promoting strains that could be applied in future microbe based bioformulations. We isolated 46 endophytic actinobacteria based on morphological characteristics of the isolates. 16S rRNA gene sequence analysis showed that the strains represented nine actinobacterial genera, *Nocardia, Amycolatopsis, Streptomyces, Pseudonocardia, Kribbella, Actinomadura, Microbispora, Rothia* and *Saccharomonospora*. *In vitro* functional characterization of the isolates for plant growth promoting (PGP) traits revealed many potent PGP isolates such as, SA1 and S43 which showed all the tested PGP traits, i.e., phosphate solubilization, indole-3-acetic acid (IAA), ammonia, siderophore and chitinase production. Out of the 46 endophytic actinobacteria isolates, 21 showed inhibition against atleast one test fungal phytopathogen and, isolates SA25 and SA29 exhibited broad spectrum antifungal activity against all the tested phytopathogens. Most of the endophytic actinobacteria isolates having antifungal activity were positive for the presence of chitinase, NRPS (Non-ribosomal peptides synthetase) or PKS-1 (Polyketide Synthase) gene, suggesting the presence of distinctive mechanisms to inhibit the growth of pathogenic plant fungi. ARDRA (Amplified Ribosomal DNA Restriction analysis) and BOX-PCR fingerprinting analysis of the potent isolates with antagonistic activity grouped the isolates into 5 and 4 separate clusters, respectively. In addition, an assessment using bonitur scale revealed the top ranked isolates based on their PGP and biocontrol traits. Further detection of IAA production by the top ranked actinobacterial isolates namely, SA1, T1LA3 and S85 by using thin-layer chromatography (TLC), high-performance liquid chromatography (HPLC) and liquid chromatography-mass spectrometry (LC-MS) was done. Endophytic actinobacteria isolates, namely, SA1, T1LA3, and SA14 were further tested for their efficacy in promoting the growth of commercial tea clones, namely, TV1, TV9, TV18, and TV22 in nursery conditions. All the endophytic isolates tested showed significant differences (*P* ≤ 0.05) in terms of plant growth promoting parameters in the treated plants compared to untreated control and may, thus be, deemed as potential candidates for application in bioformulations for tea growth.

## Introduction

Endophytic bacteria inhabit plant tissues and can boost plant growth under normal and strenuous environments by aiding plants in uptake of metabolites and by regulating phytohormone levels; improvement of plant health by aiming pathogens and pest with antagonistic metabolites, hydrolytic enzymes and nutrient limitation (Afzal et al., 2019). Endophytes are microbes found within various plant tissues, including roots, stems, leaf, flower, fruits, seeds, pollen in a symbiotic relationship without causing any apparent harm (Zhang et al., 2019). Endophytic actinobacteria are recognized as potential agents for their PGP effects and their role in nutrient uptake in host plants by producing biologically active secondary metabolites that enhance the fitness and resilience of the plants against environmental stress (Dinesh et al., 2017; Singh and Dubey, 2018). Endophytic actinobacteria have drawn a significant interest in the recent years for their ability to produce bioactive secondary metabolites with diverse function (Sathya et al., 2017).

Beneficial endophytic actinobacteria with potential biocontrol characteristics have been previously isolated from diverse plant species, such as tomato (Passari et al., 2016a), cereal crops (Patel et al., 2018), and legumes (Le et al., 2016; Vijayabharathi et al., 2018). Endophytic actinobacteria augment plant growth directly through nutrient solubilization, modulation of hormone levels, nitrogen fixation (Palaniyandi et al., 2013; Golinska et al., 2015; Figueiredo et al., 2017). Endophytic actinobacteria are also known to play a significant role in host plant protection from infectious pathogen by producing bioactive antifungal metabolites (Qin et al., 2011), polymer hydrolyzing enzymes like cellulases, siderophores and chitinase which cause degradation of fungal cell wall aiding in lysis of hyphae and limiting spore germination (Liu et al., 2017). Endophytic actinobacteria are also reported as beneficial to plant health by reducing stress hormone ethylene, with the activity of an enzyme, 1-aminocyclopropane-1-carboxylate deaminase (Yoolon et al., 2019) (ACCD) that converts ACC into ethylene.

*Camellia sinensis* L. commonly known as tea is an essential beverage crop due to its various beneficial uses, such as anti-oxidant, anti-microbial, anti-tumor and anti-inflammatory activity (Hayat et al., 2015). The primary center of origin of tea was South-East Asia adjacent to the Irrawaddy river at the conjunction of North-East India, North Burma, South-West China and Tibet provinces (Mondal et al., 2004). In North East (NE) India, Assam is the foremost producer of tea and has high socioeconomic value. The Tea plantations of NE India offer congenial environment, rendering tea crop susceptible to various fungal diseases like blister blight, black rot, branch canker, red rust, root/collar rot etc. necessitating judicious use of chemicals. Natural pathogens, including bacteria and fungi, play a significant role in the production of tea as they may have a negative influence on all stages of growth and development. In addition, the tea plants are perennial shrubs that can grow under varied climatic conditions and is constantly subjected to various environmental stresses, either from excessive soil moisture or moisture deficit (Mukhopadhyay and Mondal, 2017; Hajiboland, 2017). Drought is an important factor that affects various biological processes like membrane integrity, chlorophyll pigment content, osmotic potential adjustment, and photosynthetic activity (Anjum et al., 2011; Li et al., 2017) and subsequently limit growth and development of tea crop.

To date, rhizospheric and endophytic PGP bacteria have been explored and characterized from *C. sinensis* (Dutta et al., 2015b; Nath et al., 2015; Dutta and Thakur, 2017). However, very few data are available on tea endophytic actinobacteria especially in the North East region of India. Also, the culturable microbial diversity and the PGP potential of endophytic actinobacteria associated with wild/ornamental tea has scarcely been explored. Moreover, the naturally occurring endophytic actinobacteria which are already acclimatized to the environment of the high acidic soil of Assam, presents an underexplored source of potential PGP microbes. Thus, the present study aims largely at pursuing significant endophytic actinobacteria from a variety of wild tea species such as *C. sinensis*, *C. japonica*, *C. sasanqua*, *C. rosiflora*, *C. drupifera*, and *Eurya japonica*, which are grown and maintained in the germplasm preservation plot of Tocklai Tea Research Institute (TTRI), Jorhat, Assam, India with special reference to their disease control and plant growth promoting potential, in order to promote organic, chemical free tea cultivation. Accordingly, the present study was conducted to evaluate endophytic actinobacterial communities associated with different tissue parts of healthy tea plants using phylogenetic, functional, and molecular approach, followed by nursery experiments.

## Materials and Methods

### Collection of Samples and Endophytic Actinobacteria Isolation

Healthy wild tea species (*C. sinensis*, *C. japonica*, *C. sasanqua*, *C. rosiflora*, *C. drupifera*, and *Eurya japonica*) were collected from germplasm preservation plot of Tocklai Tea Research Institute (TTRI), Jorhat, Assam, India (26°43′45.9″N; 94°13′40.4″ E) in three seasons—winter (December—16°C temperature; 79% humidity; 35.84 mm rainfall in average), spring (April—23°C temperature; 84% humidity; 157.56 mm rainfall in average) and summer (June—27°C temperature; 91% humidity; 411.73 mm rainfall in average) (Borah et al., 2019).

Plant samples were separated into roots and leaves and washed with tap water to remove any debris. Plant samples were then surface sterilized to remove microbes present on the surface (Schulz et al., 1993). After that, surface sterilized dry plant tissues were crushed and ground aseptically in a mortar-pestle and suspended in 0.8% NaCl (1g/mL). To validate the surface sterilization procedure, aliquots of the sterile distilled water used in the final wash was spread on GLM agar media (yeast extract, 3 g; malt extract, 3 g; peptone, 5 g; starch, 10 g; agar, 20 g; distilled water, 1000mL; pH 7.4) and examined for bacterial growth after incubation at 28°C for 7 days.

If no bacterial growth was observed, the plant tissues were the spread onto five selective isolation media, namely actinomycetes isolation agar, streptomyces agar, International streptomyces project (ISP) medium 3, ISP 7, and starch casein agar (HiMedia, India). The media were supplemented with amphotericin B (75 μg/mL) and rifampicin (2.5 μg/mL) to inhibit growth of endophytic fungi and bacteria, respectively. The culture plates were incubated for 7 to 21 days at 28°C and observed daily for the growth of distinct actinobacterial morphotypic colonies with dry and rough appearance. These colonies were selected and the isolates were sub-cultured on GLM agar medium and stored in 20% glycerol at −80°C for future use.

### Morphological Characterization

The preliminary characterization of the isolated strains by cultural and morphological characteristics such as aerial and substrate mycelia color, pigment production by the isolated strains were recorded according to the International *Streptomyces* Project (Shirling and Gottlieb, 1966). Micromorphology of the strains was studied by cover slip insertion method (Williams and Cross, 1971) to observe the spore chain morphology of isolates grown on GLM media for 15 days.

### Biocontrol Activity

#### Test Fungal Pathogens

The isolates were tested for antifungal activity with different fungal phytopathogens such as *Poria hypobrunnea* (ITCC 4141), *Fusarium oxysporum* (MTCC 284), *Pestalotiopsis theae* (ITCC 6599), *Exobasidium vexans* (ITCC 938), *Rhizoctonia solani* (MTCC 4633), *Nigrospora sphaerica* (KJ767520) (Dutta et al., 2015a). The test fungal pathogens were obtained from the Microbial Type Culture Collection (MTCC), Institute of Microbial Technology, Chandigarh, India and Indian Type Culture Collection (ITCC), Indian Agricultural Research Institute, New Delhi, India.

#### Antifungal Activity

The antifungal activity of the actinobacterial isolates was assessed against six test fungal pathogens. Actinobacterial isolates were spot inoculated in GLM agar medium using spot inoculation method and incubated at 28°C for 7 days. Five days old test pathogens grown in potato dextrose broth (HiMedia) was spread on actinobacteria colonies and incubated at 28°C for 5 days. The formed antibiosis zone of inhibition was recorded, and the percentage of inhibition of mycelial growth was determined using the formula:

$$Inhibitionofmyceliagrowth\left(\%\right)=A-\frac{B}{A}\times 100$$

Where A is mycelial growth of fungal pathogen in the absence of antagonists; B is mycelial growth of fungal pathogen in the presence of antagonists.

### Plant Growth Promoting Traits

#### Indole-3-Acetic Acid Production

IAA production was assessed following the protocol of Gordon and Weber (1951) with slight modifications. The endophytic actinobacterial isolates were grown individually in GLM broth medium supplemented with L-tryptophan (1 mg/mL) at 28°C under agitated condition. After 5 days, the grown cultures were subjected to centrifugation at 3500 *g* for 7 min, and 100 μl of the supernatant was added to 96 well microplate in triplicates. 200 μl Salkowski reagent consisting of 1 ml FeCl_3_ (0.5 M) and 49 ml of 35% HClO_4_ was added to the supernatant in a ratio of 2:1 (Reagent: culture supernatant) followed by incubation at room temperature for 25 min. Positive IAA production was indicated by the development of pink or red color and absorbance was recorded at 530 nm in a multimode reader (Varioskan Flash, Thermo Fisher Scientific, United States). A standard curve was obtained using commercial IAA (Sigma–Aldrich, United States) at different known concentrations (5, 10, 20, 50, 100, and 150 μg/mL) and the concentrations of IAA produced by the actinobacteria isolates were calculated.

#### Phosphate Solubilization

Detection of phosphate solubilization was done following the protocol of Katznelson and Bose (2010) with modifications. For primary screening, actinobacterial isolates were spot inoculated on Pikovskaya’s agar medium (HiMedia, India) and incubated at 28°C for 7 days. Presence of a zone of clearance around the colonies were considered as positive isolates. For quantification, the positive actinobacterial isolates were inoculated in Pikovskaya’s broth (HiMedia, India) and incubated at 28°C in a shaker incubator for 7 days. The cultures were then centrifuged at 13,000 *g* for 10 min and triplicate aliquots of the supernatant 100 μl were added to 96 well microplate. Un-inoculated sterile media was used as negative control. Ammonium molybdate reagent containing 2.5% ammonium molybdate in 1N H_2_SO_4_ and 10% ascorbic acid was added to the supernatant in a ratio of 1:1 (Fiske and Subbarow, 1925). Optical density was obtained, and phosphate solubilizing ability was determined using a standard curve of KH_2_PO_4_ (10, 50, 100, 150, 200, 250, and 300 μg ml^–^) at 650 nm (Varioskan Flash, Thermo Fisher Scientific, United States).

#### Siderophore Production

Detection and quantification of siderophore was carried out using Chrome Azurol S assay (Schwyn and Neilands, 1987; Alexander and Zuberer, 1991). Five days old actinobacterial culture grown in GLM media were spotted on CAS (Chrome Azurol S) agar plates and incubated at 28°C for 7 days. Positive isolates were detected by the development of orange halos around the actinobacteria colonies. The quantitative measurement of siderophore production by the positive isolates detected during preliminary screening was done following the protocol of CAS-shuttle assay. Actinobacterial isolates grown in GLM broth were centrifuged at 3500 *g* for 10 min and 100 μl supernatant was added to 96 well plate along with CAS reagent in a ratio of 1:1. Uninoculated GLM broth with CAS reagent in a ratio of 1:1 was used as reference and absorbance was recorded at 630 nm (Varioskan Flash, Thermo Fisher Scientific, United States). Siderophore production was determined using the formula below (Pal and Gokarn, 2010):

$$Siderophore\left(\%\right)=\frac{A\,r-A\,s}{A\,r}\times 100$$

Where, Ar = absorbance of reference solution and As = absorbance of sample at 630 nm.

#### Ammonia Production

For estimation of ammonia production, actinobacterial isolates were grown in peptone water for 7 days at 28°C. After 7 days, the actinobacteria culture were centrifuged at 3500 *g* for 10 min. To 1 ml of the culture supernatant, 50 μl of Nessler’s reagent [7% KI; 10% HgI_2_; 50% aqueous solution of NaOH (32%)] (Cappuccino and Sherman, 2014) was added. Development of yellow to brown color indicates the presence of ammonia. The optical density at 450 nm was determined, and ammonia production by each active isolate was determined using a standard curve of ammonium sulfate solution at different known concentrations of 0.1, 0.5, 1, 1.5, 2, 2.5, 3, 3.5, and 4 umol/mL 5 (Varioskan Flash, Thermo Fisher Scientific, United States).

#### Extracellular Enzyme Production

The endophytic actinobacterial isolates were screened for the production of extracellular enzymes, namely, protease, cellulase, pectinase, chitinase and ACC deaminase. The actinobacteria isolates were grown in 50 mL of GLM broth media at 28°C for 7 days under agitated conditions. For detection of proteolytic activity, the actinobacteria isolates were spot inoculated on skim milk agar media plates comprising of (per liter) 2.5 g yeast extract, 1 g glucose, 5 g pancreatic digest of casein, 28 g skim milk powder and 15 g agar, followed by incubation at 28°C for 7 days. Positive isolates were confirmed by the presence of clear areas around the actinobacteria colony (Kazanas, 1968). Cellulase and pectinase activity was tested by spot inoculation of actinobacteria isolates on M9 minimal salt medium consisting of (per liter) 33.9 g Na_2_HPO_4_, 15 g KH_2_PO_4_, 2.5 g NaCl, 5 g NH_4_Cl, 15 g agar, 1.2 g yeast extract. For screening cellulase producing isolates, the M9 media was amended with 10 g cellulose and for pectin, the media was amended with pectin (1% w/v). After seven days of incubation at 28°C, the colonies were flooded with 1 ml of Gram’s iodine (2.0 g KI and 1.0 g iodine in 300 ml distilled water) for cellulase producing isolates detection and Congo red (0.12%) for pectinase producing isolates detection. Presence of clear zone surrounding the colonies were considered positive (Teather and Wood, 1982; Kasana et al., 2008). Chitinase production was assessed by spot inoculating actinobacteria isolates on M9 minimal salt agar plates containing 1% w/v colloidal chitin and 1.2 g yeast extract followed by incubation for 7 days at 28°C. Development of zone of clearance around the colonies were considered as positive for chitinase production (Hsu and Lockwood, 1975). Qualitative assay for ACC deaminase activity was performed by spot inoculation of actinobacterial isolates in Dworkin and Foster (DF) salt medium (Dworkin and Foster, 1958) modified with 3 mM filter sterile, ACC (Onofre-Lemus et al., 2009). Actinobacteria isolates were grown in 50 ml of DF media amended with 3 mM ACC and incubated at 28 ± 2°C under agitated conditions at 200 rev min^–1^ for 5–7 days. DF agar plates were then spread with 3mM ACC solution and allowed to dry fully before actinobacterial spot inoculation. The inoculated plates were incubated at 28 ± 2°C for 5-days and actinobacterial growth was observed.

#### Assessment of PGP and Biocontrol Traits

The PGP traits, namely, IAA production, phosphate solubilization, ammonia production, nitrogen fixation, chitinase activity and siderophore production by the endophytic actinobacterial isolates were analyzed and represented as Venn diagram using Vennture software (Martin et al., 2012). Similarly, the biocontrol traits profile of all the endophytic actinobacterial isolates were also represented as Venn diagram using Vennture software (Martin et al., 2012). To select actinobacterial isolates with superior PGP potential, a bonitur scale was generated and applied for the evaluation of PGPR and biocontrol traits similar to that of Passari et al. (2016b). In this scale, points were allocated for each *in vitro* PGP and biocontrol traits tested. The highest possible bonitur score for the PGP traits is 29 points. A maximum of three points were given to antifungal activity against six tested pathogens depending on percentage of inhibition, leading to a sum of 18 points. IAA production and phosphate solubilization was each given maximum three points depending on the strength of the PGP traits. Siderophore, ammonia production, chitinase and ACC deaminase were assigned one point each.

### Phosphate Solubilization Efficiency of the Endophytic Actinobacterial Isolates

For assessment of phosphate solubilization at different initial pH (alkaline, neutral and acidic pH), three actinobacterial isolates were selected on the basis of Bonitur ranking. The three isolates, SA1, SA14 and S85 were quantitatively tested using colorimetric assay for their efficiency to solubilize phosphate at different initial pH of 6.0, 7.0 and 8.0 (Marra et al., 2015).

The actinobacterial isolates were inoculated in Pikovskaya’s broth (HiMedia, India) and incubated at 28°C in a shaker incubator for 7 days. The cultures were then centrifuged at 13,000 *g* for 10 min and triplicate aliquots of the supernatant 100 μl were added to 96 well microplate. Un-inoculated sterile media was used as negative control. Ammonium molybdate reagent containing 2.5% ammonium molybdate in 1N H_2_SO_4_ and 10% ascorbic acid was added to the supernatant in a ratio of 1:1 (Fiske and Subbarow, 1925). Optical density was obtained, and phosphate solubilizing ability was determined using a standard curve of KH_2_PO_4_ (10, 50, 100, 150, 200, 250, and 300 μg ml^–^) at 650 nm (Varioskan Flash, Thermo Fisher Scientific, United States).

### Extraction, Purification, and Determination of IAA

#### Extraction of Crude IAA

For determination of IAA production, isolates SA1, T1LA3 and S85 were selected on the basis of Bonitur assessment. Actinobacterial colony of these IAA producing isolates were inoculated into 200 ml GLM broth amended with L-tryptophan (1 mg/mL) and incubated at 28°C for 7 days in an orbital-shaking incubator. Bacterial supernatant was then separated from the bacterial cells by centrifugation at 10,000 *g* for 20 min, followed by filtration using a Whatman, 0.2 μm filter paper to acquire a cell-free supernatant. The supernatant was acidified to pH 2.5–3.0 using 1 N HCl, extracted with ethyl acetate in a ratio of 1:1 (v/v) by vigorous mixing of the cell free supernatant and separating the organic phase in a separatory funnel (Bharucha et al., 2013). The extracted ethyl acetate fraction was evaporated in a rotary evaporator (Rotavapor R-210, Buchi, Switzerland) at 50°C. The crude extract was dissolved in 2 ml of methanol and kept at 4°C for further tests.

#### Detection of IAA by Thin Layer Chromatography

Extracts of crude IAA were plated on pre-coated silica gel 60 F_254_ TLC plates (Merck, Germany) along with standard IAA (Sigma–Aldrich, United States). The IAA standard was prepared at a concentration of 0.5 mg/mL in methanol and TLC was performed with a mobile phase of acetone: ethyl acetate: ethanol: water (3:5:1:1 v/v/v/v) (Ahmad et al., 2005). TLC plates were dried and spots with identical RF values to standard IAA were observed under UV light (254 nm) and developed by spraying with Salkowski reagent.

#### Detection of IAA by High Performance Liquid Chromatography

HPLC analysis of the crude ethyl acetate extract was carried out on a Waters HPLC, Breeze 2 (Massachusetts, United States) using a C18 analytical column (4.6 mm × 250 mm, 5 μm) (ODS2, Waters spherisorb). The column temperature was maintained at 25°C and methanol and 1% acetic acid (50:50 v/v) was used as the mobile phase at a flow rate of 1 mL/min with an injection volume of 20 μL (Myo et al., 2019). Detection was monitored at 254 nm and 280 nm, and data were evaluated using Breeze 2 software by comparing with the elution profiles of standard IAA (0.5 mg/mL) injected separately.

#### Detection of IAA by Liquid Chromatography–Mass Spectrometry

An Ultimate 3000 rapid separation LC (Dionex Inc., Sunnyvale, CA, United States) coupled to a Thermo Exactive plus Orbitrap triple quadrupole Mass Spectrometer (Thermo Fisher Scientific, United States) equipped with an electrospray ionization (ESI) interface was used to detect IAA in crude ethyl acetate extract. The chromatographic separation of each sample and standard IAA (0.5 mg/mL) was on a C18 column (Hypersil gold, Dim. 150 mm × 2.1 mm, 1.9 μm) (Thermo Fisher Scientific, United States) with 0.1% formic acid in water (mobile phase A) and methanol (mobile phase B) was used and the column temperature was set at 25°C. Gradient elution at a flow rate of 0.300 mL/min was performed. The gradient program was as follows: 0–2.5 min from 20 to 40% B; 2.5–4.0 min from 40 to 50% B; 4.0–5.0 min from 50 to 98% B; 5.0–6.0 min 98% A to 2% B; and 6.0–8.0 min 20% A to 80% B. The total run time was 8.0 min, and a volume of 10 μL was injected onto the Hypersil C18 gold column by an RS-3000 autosampler (Dionex Inc., Sunnyvale, CA, United States). ESI-MS was performed in both the modes with a spray voltage of 4 kV, capillary temperature at 270°C; auxiliary gas heater temperature, 45°C, auxiliary gas flow rate at 6 (arbitrary units) and a full scan data were obtained by scanning from m/z 50–750. The chromatograms for each sample were recorded using a PDA detector and data acquisition and processing were done using the Xcalibur software (Thermo Fisher Scientific, United States).

#### Biochemical Profile of Top Ranked Isolates

Biochemical characterization of the highest bonitur ranked three isolates was performed with a number of tests, including carbohydrate utilization, β-galactosidase activity, esculin hydrolysis, citrate and malonate utilization. The tests were performed using KB009 HiCarbohydrate kit (HiMedia, India). Actinobacterial isolates grown in GLM broth were centrifuged at 3500 *g* and the cells were washed and resuspended in saline solution (0.9% NaCl). Each of the wells of the kit was inoculated with 50 μl of the actinobacterial suspension by surface inoculation method and incubated at 28°C for 4–7 days. Changes in the color of the medium were recorded.

#### Fungal Hyphal Morphology of *P. hypobrunnea* as Affected by Isolate T1LA3

Test fungal pathogen, *P. hypobrunnea* was co-inoculated with endophytic actinobacteria isolate, T1LA3 in PDA media at 28°C for 10 days. Fungal mycelia of plant pathogen, *P. hypobrunnea* grown on PDA plate along with actinobacterial isolate, T1LA3 from the edge of zone of inhibition were mounted on glass slides for lactophenol cotton blue staining and observed under a light microscope (100X with oil suspension; Motic BA410).

### Phylogenetic Characterization

#### Genomic DNA Extraction and 16S rRNA Gene Amplification

Genomic DNA was extracted from the actinobacterial strains using Nucleopore Fungus/bacteria kit (Genetix, India). 16S rRNA gene amplification was done using universal primers 27F (5′-AGA GTT TGA TCC TGG CTC AG-3′) and 1492 R (5′-GGT TAC CTT GTT ACG ACT T-3′) as previously described (Hongoh et al., 2003) in 50μl reaction mixture containing 0.2μM of each primer, 0.2 mM of each dNTP, 1X Taq buffer, 1.5mM MgCl_2_, 1U TaqDNA polymerase (TaKaRa Bio Inc., Japan) and 50 ng of template DNA. The PCR cycle was carried out in a Mastercycler nexus PCR system (Eppendorf, Germany) and the cycle consisted of initial denaturation (94°C for 5 min), 35 cycles of denaturation (94°C for 30 s, 52°C for 30 s, 72°C for 1 min), annealing (72°C for 1 min) and final extension (72°C for 1 min). Then, the amplified products were subjected to gel electrophoresis using a 1.5% agarose gel stained with EtBr (10 μg/mL) at 70V for 45 min and visualized in a gel documentation system (Bio Rad ChemiDoc XRS +). The PCR amplified products were then sent to DNA sequencing services (1st BASE DNA Sequencing Services, Base Asia) for Sanger sequencing.

#### Amplified Ribosomal DNA Restriction Analysis

The potent endophytic actinobacterial isolates showing positive antifungal activity were subjected to Amplified ribosomal DNA restriction analysis (ARDRA). The amplified 16 S rRNA were subjected to restriction digestion using 2 U of each of the enzymes *Hin*fI, *Kpn*I, *Sau*3A1, *Hae*III, and *Hind*I (New England Biolabs, United Kingdom) as manufacturer’s recommendation. The digestion was done in 25 μl reaction mixture and incubated at 37°C for 2 h followed by heat inactivation at 70°C for 10 min. Each digested sample mixed with 4 μl 6X loading dye was subjected to 2% agarose gel electrophoresis at 70V for 3 h along with 100 bp DNA ladder and visualized in a gel documentation system (ChemiDoc XRS +, Bio-Rad, United States) and analyzed by NTSYS-pc version 2.02. software (Applied Biostatistics Inc., New York).

#### BOX-PCR

The potent endophytic actinobacterial isolates showing positive antifungal activity were subjected to genotypic analysis by rep-PCR fingerprinting using BOX-A1R primer (5′-CTACGGCAAGGCGACGCTGACG-3′) (Smith et al., 2001). PCR amplification was carried out in 25 μl volume containing 1X Taq buffer, 0.1 U Taq polymerase, 2.5 mM each deoxynucleotide triphosphate (dNTP), 1.5 mM MgCl_2_, 2.5 mM of BOX-A1R primer and 10 ng DNA template under the following conditions: 1 min at 94°C, 35 cycles at 94°C, 55°C for 1 min each and 72°C for 1.5 min and final extension at 72°C for 7 min (Lee et al., 2014). The PCR products along with 100 bp DNA ladder (TaKaRa Bio Inc., Japan) were subjected to 2% agarose gel electrophoresis and visualized using a gel documentation system (ChemiDoc XRS +, Bio-Rad, United States) followed by analysis using Phoretix 1D Pro gel analysis software (TotalLab Ltd., Newcastle upon Tyne, United Kingdom).

#### Phylogenetic Analysis

The 16 S rRNA gene sequence obtained was compared against DNA Databank of Japan (DDBJ) and NCBI via BLAST analysis to retrieve similar sequences (Zhang et al., 2000). A Neighbor joining (NJ) phylogenetic tree was constructed (Saitou and Nei, 1987) using MEGA 7.0 (Kumar et al., 2016) with bootstrap analysis based on 1000 replicates (Felsenstein, 1985). The evolutionary distance was measured using the p-distance method (Nei and Kumar, 2000). Additionally, the banding pattern obtained in ARDRA was scored to generate a dendrogram using the NTSYSpc version 2.02 software. The phylogenetic relationship was determined according to the method of unweighted pair group method with arithmetic mean (UPGMA) (Sokal and Michener, 1958) using DICE similarity coefficient, all the strains were grouped into different phylotypic groups. Similarly, the fingerprints generated during the BOX-PCR were analyzed using Phoretix 1D Pro gel analysis software (TotalLab Ltd., United Kingdom) to obtain a dendrogram according to UPGMA using DICE similarity coefficient.

### Molecular Characterization

#### Chitinase Gene

The isolates which were positive for chitinase activity in plate assay were subjected to chitinase gene amplification using PCR. Chitinase gene, 18 family PCR amplification was carried out using primers GA1F and GA1R as previously reported (Williamson et al., 2000). The PCR reaction mixture (10 μl total volume) contained 1X Taq buffer, 0.2 μM of each primer, 125 μM each dNTP and 0.5 U of Taq polymerase and 10 ng template DNA. The PCR amplification cycle consisted of 5 min at 94°C, 35 cycles of 1 min at 94°C and 30 s at 60°C, and a final extension of 7 min at 72°C and was carried out in a Mastercycler nexus PCR system (Eppendorf, Germany). The PCR products were subjected to gel electrophoresis using 1.5% agarose gels stained with EtBr (10 μg/mL) at 70V for 45 min along with 100bp DNA ladder and visualized using a gel documentation system (ChemiDoc XRS +, Bio-Rad, United States).

#### Biosynthetic Gene Clusters

NRPS, PKS I and PKS II gene amplification were carried out using degenerate primers (Ayuso-Sacido and Genilloud, 2005). The PCR cycle consists of 5 min at 95°C and 35 cycles of 30 s at 95°C, 2 min at 55°C for K1F/M6R, 59°C for A3F/A7R or 58°C for K1F/M6R and A3F/A7R and 4 min at 72°C, followed by 10 min at 72°C. PCR amplified products were analyzed by 1% agarose gel electrophoresis stained with ethidium bromide and visualized using a gel documentation system (ChemiDoc XRS +, Bio-Rad, United States).

### Plant Growth Promoting Experiment in Nursery Condition

#### Plant Sample

Tea a woody perennial slow growing tree crop, which is commonly propagated through vegetative propagation. Tea crop is mainly propagated through vegetative propagation for maintaining quality in commercial cultivation. The Tocklai tea Research Institute, Jorhat, Assam, India have until now released 33 vegetatively propagated clones (TV series) which are either standard or yield clones for the purpose of commercial cultivation especially in Northeast India. In this study, we selected two standard clones (TV1 and TV9) and two yield clones (TV18 and TV22) available at Betali Tea estate, Assam, India for the PGP experiment in nursery conditions. The TV clones are grown for about 6–7 months of growth in polythene sleeves before they can be transferred to the field. The tea saplings are grown and maintained in polythene sleeves following the standard protocol of sleeve preparation for vegetative propagation of tea (Field Management Notes, TTRI, Jorhat). The commercial tea is grown through vegetative propagation using cuttings. The cuttings are first treated in 0.1% ZnSO_4_ solution and planted in nursery beds. After root formation (8–12 weeks), the cuttings are transferred to sleeves containing fine tilth soil obtained by sieving with G1 wire mesh no. 4 and mixed with phosphate at 500 g/m^3^ of soil. The soil used in the sleeves are sandy loam soils maintained within the pH range of 4.5 to 5.5 (Field Management Notes, TTRI, Jorhat). The size of the sleeve is 17.7 cm lay flat, 25 cm long and 150 gauge thick. The cuttings are grown in these sleeves for about 5–6 months and then transferred to the garden (Borah et al., 2019). Four different types of healthy 3–4 months old TV clones, TV1, TV9, TV18 and TV22 were selected for *in vivo* PGP experiment and procured from Betali Tea estate, Assam.

#### Preparation of Actinobacterial Inoculum and Treatment

Three potent actinobacterial strains based on the ranking derived from bonitur assessment were considered for the PGP experiment in nursery conditions. The pure actinobacteria isolates were individually inoculated in GLM culture broth and grown at 28°C for 7 days under agitated conditions. The culture was centrifuged at 8000 *g* for 5 min and the cell pellet was resuspended in phosphate buffer saline (pH 7.4). The cell density was adjusted to 10^8^ CFU/mL at 610 nm using a spectrophotometer (Singh et al., 2016).

Five treatments including (1) untreated tea saplings as control, (2) treatment 1 (SA1), (3) treatment 2 (T1LA3), (4) treatment 3 (SA14) (4) consortia (SA1 + T1LA3 + SA14) (five biological replicates for each treatment) were applied to the saplings. Unsterilized soil was used in the experiment to determine the effects of these bacterial strains on tea clones in competition with the naturally occurring soil microbes. The tea clones were treated with 10 ml cell suspension by soil drenching at one-month intervals for three months and watered with sterile distilled water daily (Liotti et al., 2018; Zhao et al., 2018). All vegetative parameters such as shoot length, root length, shoot fresh and dry weight and root fresh, dry weight, chlorophyll content and soil parameter analysis were recorded and compared to control (Dutta et al., 2015b; Dutta and Thakur, 2017).

#### Chlorophyll Estimation

The chlorophyll content in leaf tissues of the treated tea clones with reference to untreated tea clones was determined following the method of Hiscox and Israelstam (2011). Leaf tissue were washed in tap water followed by distilled water and cut into fractions. 100 mg of the cut tissue was placed in vials containing 7 ml of DMSO (HiMedia) and chlorophyll was extracted into the fluid by incubating the vials for 1 h at 65°C without grinding. The extract was transferred to a graduated tube and volume was adjusted to 10 ml with DMSO and absorbance was recorded at 645 nm and 663 nm. The chlorophyll content was calculated using the following formula:

C⁢h⁢l⁢a=11.75×A⁢663-2.35×A⁢645

C⁢h⁢l⁢b=18.61×A⁢645-3.96×A⁢663

#### Soil Parameter Analysis

Soil samples from treated and untreated control were collected from 0 to 15 cm, during harvest and sent to Sigma Test and Research Centre, Delhi for soil chemical analysis (Potassium,% by mass; Organic Matter,% by mass Total Phosphorus,% by mass; Available Phosphorus,% by mass; Total Nitrogen,% by mass; Available Nitrogen,% by mass; Sulfur (as So_4_),% by mass; Calcium,% by mass; Magnesium,% by mass; Water holding capacity,% by mass; Manganese, mg/kg, Iron, mg/kg; Copper, mg/kg; Zinc, mg/kg; Molybdenum, mg/kg, Boron, mg/kg).

### Statistical Analysis

The data generated through the nursery trial for different growth parameters were subjected to statistical analysis. A two-way Analysis of variance (ANOVA) using the triplicate value between different PGP parameter of treated tea plants compared to the control group for all the clones were assessed. *P* ≤ 0.05 was considered statistically significant. Also, principal component analysis (PCA) was done based on the eigen values of a matrix on the PGP datasets to study the correlation between growth parameters and bacterial treatment in the PGP experiment. Furthermore, fold change analysis was done to assess the total value difference between treatment and control averages and the output values are generated in a log_2_ scale.

## Results

### Isolation of Endophytic Actinobacteria

A total of 46 endophytic actinobacteria were isolated from roots and leaf tissue of five different *Camellia* spp. and related genera. Morphological characterization of the isolates was recorded (Table 1 and Supplementary Figure 1).

**TABLE 1:** Morphological characterization of endophytic actinobacteria associated with tea.

**Sl. No.**	**Isolate code**	**Colony morphology**	**Aerial mycelia color**	**Substrate mycelia color**	**Diffusible pigment**	**Color series**
1	SA1	Irregular, Raised	Brown	Yellow	ND	Brown
2	SA2	Circular, Flat	Red	Red	Red	Red
3	T1LA3	Curled, Convex	White	Orange	Light Pink	White
4	SA16	Circular, Crateriform	White	Off White	ND	White
5	SA6	Irregular, Raised	White	Yellow	ND	White
6	CSLA2	Circular, Crateriform	Yellow	Yellow	ND	White
7	CJRA1	Irregular, Raised	Gray	Brown	ND	Gray
8	S40	Circular, Umbonate	Off White	Off White	ND	White
9	SA21	Circular, Convex	Cream	Cream	ND	White
10	SA5	Irregular, Umbonate	Black	Dark Gray	Yellow	Gray
11	CSLA5	Irregular, Flat	Yellow	Yellow	ND	White
12	S51	Irregular, Flat	White	Gray	ND	Gray
13	S62	Irregular, Flat	Brown	Brown	ND	Brown
14	S28	Circular, Flat	Light Pink	White	ND	White
15	T1LA5	Irregular, Raised	Gray	Black	ND	Gray
16	S39	Punctiform	White	Yellow	ND	White
17	SA13	Umbonate, Raised	Brown	Brown	ND	White
18	SA14	Circular, Umbonate	White	Brown	ND	White
19	SA9	Irregular, Raised	Gray	Black	ND	Gray
20	S41	Irregular, Raised	Brown	Brown	ND	Brown
21	S85	Circular, Umbonate	White	Brown	ND	White
22	S42	Irregular, Raised	Off White	Off White	ND	White
23	SA10	Irregular, Raised	White	Gray	ND	White
24	SA20	Circular, Flat	Yellow	Yellow	ND	White
25	S84	Irregular, Umbonate	Light Pink	Peach	ND	White
26	CJRA5	Circular, Flat	Light Pink	Off White	ND	Variable
27	CJRA2	Irregular, Raised	White	Off White	ND	White
28	CSLA7	Circular, Crateriform	Brown	Brown	ND	Variable
29	CRRA1	Irregular, Umbonate	Brown	Brown	Brown	Brown
30	SA7	Irregular, Flat	Pink	Pink	Pink	Pink
31	SA8	Circular, Flat	Gray	Brown	Brown	Gray
32	SA4	Irregular, Raised	White	Yellow	Yellow	White
33	S43	Circular, Flat	Gray	Black	ND	Gray
34	S72	Irregular, Raised	Gray	Yellow	ND	White
35	CSR1	Circular, Raised	Off White	Off White	ND	White
36	SA27	Circular, Crateriform	Peach	Peach	ND	Orange
37	SA35	Circular, Raised	White	Cream	ND	White
38	SA17	Circular, Flat	Gray	Gray	ND	Gray
39	SA12	Irregular, Raised	Peach	Brown	ND	Variable
40	SA29	Circular, Crateriform	White	Yellow	ND	White
41	SA3	Undulate, Flat	White	Orange	Yellow	White
42	SA25	Irregular, Flat	Brown	Brown	Brown	Brown
43	CSR3	Irregular, Convex	Light Yellow	Light Yellow	ND	Yellow
44	CSLA6	Irregular, Umbonate	Brown	Brown	Brown	Brown
45	CSR4	Irregular, Raised	Gray	Dark Pink	Pink	Gray
46	SA11	Circular, Crateriform	Light Pink	Yellow	ND	White

*ND, not detected.*

### Plant Growth Promoting Traits

**FIGURE 1:**
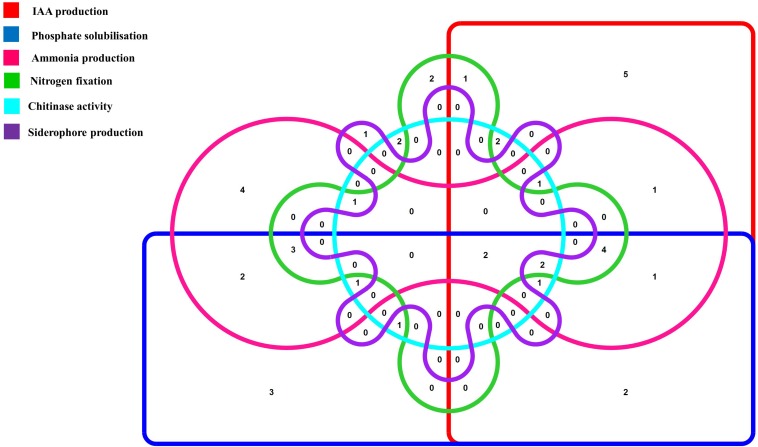
Venn diagram representation of plant growth promoting endophytic actinobacterial isolates showing different PGP traits. The Venn diagram shows the allocation of 46 PGP endophytic actinobacterial isolates into 6 profiles representing 6 PGP traits.

To further characterize the endophytic actinobacterial isolates, their plant growth promoting (PGP) traits were tested (Supplementary Figure 2). The PGP traits of the endophytic actinobacteria are summarized in Table 2. Out of 46 strains, 21 (45.6%) solubilized phosphate at levels ranging from 59.1 ± 1.6 to 277.5 ± 4.8 μg/mL and SA16 solubilized the highest amount of phosphate (277.5 ± 4.8 μg/mL) among all the isolates. 23 isolates (50%) out of 46 isolates produced IAA levels in the range 4.4 ± 0.1 to 46.5 ± 0.2 μg/mL among which CJRA1 produced the highest amount of IAA (46.5 ± 0.2 μg/mL). Ammonia production was shown by 22 isolates in the range 1.3 to 6.5 μmol ml^–1^. T1LA3 isolate showed the highest amount of ammonia production of 6.5 μmol ml^–1^. 15 (32.6%) isolates out of the total isolated endophytic actinobacterial isolates were able to grow on N_2_ free medium. Siderophore production was showed by 3 isolates out of the total 46 isolates. Two isolates, SA1 and S43 showed all the tested PGP traits, i.e., phosphate solubilization, IAA, ammonia, siderophore and chitinase production (Figure 1).

**TABLE 2:** Plant growth promoting and antifungal characteristics of endophytic actinobacteria isolated from tea plants.

**Sl. no.**	**Strain code**	**IAA (μg ml**^–^**^1^)**	**P solubilization (μg ml**^–^**^1^)**	**Ammonia (μmol ml**^–^**^1^)**	**N_2_ fixation**	**Siderophore (%)**	**Inhibition of mycelia growth (%)**					
							
							**1**	**2**	**3**	**4**	**5**	**6**
1	**SA1**	19.0 ± 4.0	141.1 ± 17.2	2.9 ± 0.5	+	30.5 ± 0.6	−	−	−	75 ± 1.3	85.7 ± 1.3	62.7 ± 1.2
2	**SA2**	14.8 ± 0.5	107.8 ± 4.6	5.5 ± 0.7	+	−	−	62.5 ± 1.0	−	−	−	78 ± 2.1
3	**T1LA3**	23.2 ± 0.2	−	6.5 ± 0.8	−	12.1 ± 0.3	86.8 ± 1.1	−	−	82.5 ± 1.5	57 ± 1.1	76.7 ± 1.6
4	**SA16**	40.7 ± 0.7	277.5 ± 14.5	2.1 ± 1.0	+	−	−	−	−	−	−	−
5	**SA6**	9.2 ± 0.2	−	−	+	−	75 ± 1.8	−	−	50 ± 1.2	−	44 ± 2.0
6	**CSLA2**	4.4 ± 0.4	−	−	−	−	−	−	−	−	−	−
7	**CJRA1**	46.5 ± 0.7	−	−	−	−	54.5 ± 1.19	−	−	−	−	−
8	**S40**	7.1 ± 0.1	171.9 ± 3.0	3.0 ± 0.8	−	−	34 ± 1.07	−	−	−	−	−
9	**SA21**	−	−	5.5 ± 0.4	+	−	−	−	90 ± 0.9	−	−	−
10	**SA5**	−	171.7 ± 11.8	−	−	−	−	−	−	−	−	−
11	**CSLA5**	−	142.8 ± 7.4	2.1 ± 1.0	+	−	−	−	−	−	−	−
12	**S51**	−	−	−	−	−	−	−	−	−	−	−
13	**S62**	22.9 ± 0.3	127.9 ± 20.0	5.3 ± 0.4	+	−	65 ± 1.5	−	−	25 ± 0.5	40 ± 1.1	25 ± 0.4
14	**S28**	−	−	3.3 ± 0.5	−	−	−	−	−	−	−	72 ± 1.9
15	**T1LA5**	−	−	−	−	−	−	−	−	−	−	−
16	**S39**	19.2 ± 0.4	216.2 ± 10.7	2.5 ± 1.1	+	−	−	−	−	−	−	−
17	**SA13**	−	153.2 ± 7.7	−	−	−	−	−	−	62.5 ± 2.4	−	50 ± 2.3
18	**SA14**	−	219.3 ± 0.8	3.6 ± 0.5	−	−	30 ± 0.2	80 ± 2.5	−	−	62.8 ± 2.5	76.7 ± 1.6
19	**SA9**	−	−	−	−	−	−	−	−	−	−	−
20	**S41**	7.1 ± 0.2	188.6 ± 20.1	1.4 ± 0.1	+	−	−	−	−	−	−	−
21	**S85**	39.0 ± 0.8	187.2 ± 14.8	−	−	−	65.9 ± 1.4	35.4 ± 1.7	−	−	−	53.4 ± 1.8
22	**S42**	17.6 ± 0.4	59.1 ± 5.0	−	−	−	−	−	−	−	−	−
23	**SA10**	−	−	−	−	−	−	−	−	−	−	−
24	**SA20**	−	−	−	−	−	−	−	−	−	−	−
25	**S84**	5.6 ± 0.3	190.9 ± 19.9	4.3 ± 0.3	+	−	25 ± 1.2	−	−	−	−	−
26	**CJRA5**	−	−	−	−	−	−	−	−	−	−	−
27	**CJRA2**	−	−	−	+	−	−	−	−	−	−	−
28	**CSLA7**	−	−	−	−	−	−	−	−	−	−	−
29	**CRRA1**	7.0 ± 0.2	−	−	−	−	−	35.4 ± 1.0	−	−	−	−
30	**SA7**	−	256.4 ± 13.7	4.4 ± 0.6	+	−	−	−	−	−	−	−
31	**SA8**	18.8 ± 0.7	−	−	−	−	−	35.4 ± 1.6	−	−	−	65.1 ± 0.9
32	**SA4**	10.5 ± 1.5	−	1.3 ± 0.2	−	−	−	−	−	−	−	−
33	**S43**	19.4 ± 0.4	132.7 ± 10.9	4.7 ± 0.6	+	27.0 ± 0.9	−	41.9 ± 2.9	−	−	−	62.5 ± 1.4
34	**S72**	−	135.1 ± 18.0	−	−	−	−	−	−	−	−	−
35	**CSR1**	10.6 ± 0.6	183.4 ± 12.6	2.5 ± 0.3	+	−	−	−	−	−	−	−
36	**SA27**	12.6 ± 2.3	−	3.3 ± 0.4	−	−	54.5 ± 1.3	19 ± 0.8	43.3 ± 2.3	−	−	−
37	**SA35**	12.8 ± 0.6	−	−	−	−	−	−	−	−	−	−
38	**SA17**	−	−	−	−	−	−	−	−	−	−	−
39	**SA12**	−	201.8 ± 15.9	4.4 ± 0.5	−	−	65.1 ± 1.7	−	−	57.5 ± 2.9	−	−
40	**SA29**	5.0 ± 0.7	−	5.7 ± 0.9	−	−	59 ± 1.3	41.9 ± 1.0	40 ± 1.1	22.5 ± 0.3	42.8 ± 3.1	37.2 ± 2.1
41	**SA3**	−	−	−	−	−	−	−	−	−	−	−
42	**SA25**	7.1 ± 0.7	213.7 ± 16.5	2.3 ± 0.4	−	−	54.5 ± 1.9	25.8 ± 1.6	46.6 ± 2.1	50 ± 1.2	51.5 ± 2.3	32.5 ± 0.7
43	**CSR3**	−	−	−	−	−	−	−	−	−	−	−
44	**CSLA6**	−	247.9 ± 5.9	2.3 ± 0.6	+	−	−	38.7 ± 0.8	−	−	−	−
45	**CSR4**	−	−	−	−	−	−	−	−	−	−	−
46	**SA11**	−	−	−	−	−	−	−	−	−	−	−

*The values are ± Standard error (*n* = 3). (1) + Growth or PGP activities detected; - No growth, no inhibition or no PGP activity detected. IAA, Indole acetic acid; P, Phosphate; N_*2*,_ Nitrogen. (2) Test pathogens: 1: *P. hypobrunnea* (ITCC 4141); 2: *F. oxysporum* (MTCC 284); 3: *P. thaea* (ITCC 6599); 4: *E. vexans* (ITCC 938), 5: *R. solani* (MTCC 4633); 6: *N. sphaerica* (KJ767520).*

### Extracellular Enzyme Production

Investigation of hydrolytic enzyme producing ability of the isolates indicated that protease producers were more prevalent among all the isolates (16 isolates, 34.7%), followed by ACC deaminase producers (13 isolates, 28.2%). Cellulase and pectinase production was shown by 12 and 10 strains, respectively. In addition, 12 (26.0%) isolates out of the total isolated endophytic actinobacterial isolates showed chitinolytic activity (Table 3).

**TABLE 3:** Extracellular hydrolytic enzyme production by endophytic actinobacteria associated with tea.

**Sl. no.**	**Strain code**	**Protease**	**Pectinase**	**Cellulase**	**ACC deaminase**	**Chitinase**
1	SA1	+	−	−	+	+
2	SA2	+	+	+	−	+
3	T1LA3	−	−	+	−	−
4	SA16	−	+	−	−	−
5	SA6	+	−	−	+	−
6	CSLA2	−	−	−	+	−
7	CJRA1	−	−	−	−	+
8	S40	−	+	+	−	+
9	SA21	+	−	−	−	+
10	SA5	+	−	−	−	−
11	CSLA5	−	−	−	−	−
12	S51	+	+	+	+	−
13	S62	−	+	+	−	+
14	S28	−	+	+	−	−
15	T1LA5	−	−	−	−	−
16	S39	−	−	+	−	−
17	SA13	−	−	−	−	+
18	SA14	−	−	−	−	−
19	SA9	−	−	−	−	−
20	S41	+	−	−	+	−
21	S85	+	+	−	+	−
22	S42	−	−	−	+	−
23	SA10	−	−	−	−	−
24	SA20	−	−	−	−	−
25	S84	+	+	−	−	−
26	CJRA5	−	−	−	−	−
27	CJRA2	−	−	+	−	−
28	CSLA7	−	−	−	−	−
29	CRRA1	−	−	−	−	+
30	SA7	+	−	−	+	−
31	SA8	−	−	+	−	+
32	SA4	−	−	−	−	−
33	S43	−	+	+	−	+
34	S72	−	−	−	+	−
35	CSR1	+	−	−	−	−
36	SA27	+	+	−	+	+
37	SA35	−	−	−	−	−
38	SA17	+	−	−	−	−
39	SA12	+	−	−	−	+
40	SA29	−	−	−	+	−
41	SA3	−	−	+	+	−
42	SA25	+	−	+	+	−
43	CSR3	−	−	−	−	−
44	CSLA6	+	−	−	−	−
45	CSR4	−	−	−	−	−
46	SA11	−	−	−	−	−

*+ Growth or enzyme activity detected; - No growth, or no enzyme activity detected.*

### Biocontrol Traits

**FIGURE 2:**
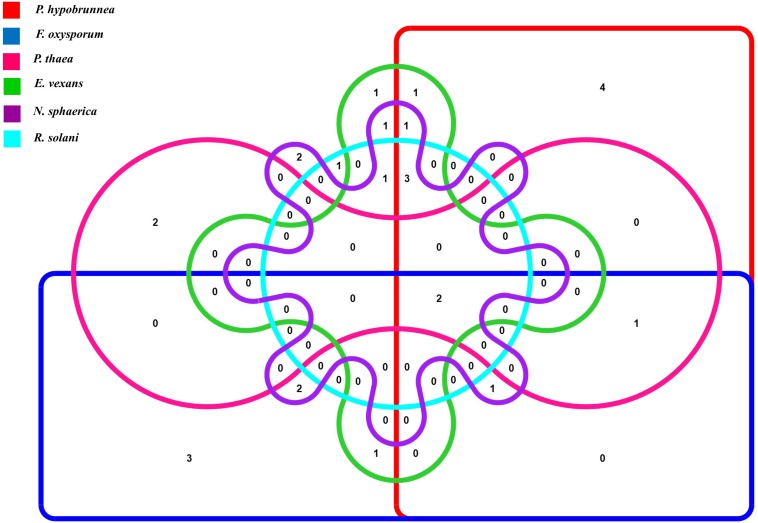
Venn diagram representation of antifungal traits of 46 endophytic actinobacteria isolates showing 6 profiles representing the 6 tested tea phytopathogens.

The antifungal activities of all the strains were tested against six fungal phytopathogens 21 (45.6%) strains showed antifungal activity against at least one of the test pathogens. Altogether, 12 strains inhibited the growth of *P. hypobrunnea* and *N. sphaerica* each. The growth of *E. vexans* and *F. oxysporum* were inhibited by 10 and 9 strains, respectively. The growth of fungal phytopathogen, *P. theae* was inhibited by only 4 isolates. Only two isolates, SA25 and SA29 exhibited broad spectrum antifungal activity against all the tested phytopathogens (Figure 2). In addition, the highest percentage of inhibition was shown by SA21 against *P. theae* (90%) followed by T1LA3 against *P. hypobrunnea* (86.8%).

### Analysis of PGP and Biocontrol Traits

An assessment using bonitur scale was done in order to select the most potent endophytic actinobacteria having PGP as well as biocontrol traits (Passari et al., 2016b). In this scale, points were assigned to each PGP and biocontrol traits tested and the maximum possible score is 29. The maximum point is three given to antifungal activity against each tested pathogen depending on percentage of inhibition, leading to a sum of 18 points. IAA production and phosphate solubilization was each given maximum three points depending on the strength of the PGP traits. Siderophore, ammonia production, chitinase and ACC deaminase were assigned one point each. The top three ranking isolates, SA1, T1LA3, and SA14 were considered for further testing of the efficacy of the endophytic actinobacterial isolates on tea plants in nursery condition (Table 4).

**TABLE 4:** The most potent isolates based on Bonitur ranking of endophytic actinobacteria associated with tea with PGP and biocontrol traits.

**Sl. No.**	**Strain code**	**Antagonistic activities**						**Antifungal mechanisms**		**PGP traits**					**Total ass. (29)^*n*^**	**Ranking**
						
		**Ph^a^**	**Fo^b^**	**Pt^c^**	**Ev^d^**	**Rs^e^**	**Ns^f^**	**Chi^g^**	**Sid^h^**	**IAA^i^**	**PS^j^**	**AM^k^**	**ACC^l^**	**Sid^m^**		
1	SA1	0	0	0	3	3	2	1	1	2	2	1	1	1	17	1^*st*^
2	T1LA3	3	0	0	3	2	3	0	1	2	0	1	1	1	17	1^*st*^
3	SA14	1	0	0	3	2	3	1	0	0	3	1	0	0	14	2^*nd*^
4	SA12	2	0	0	2	0	0	1	0	0	3	1	1	1	11	3^*rd*^
5	S85	2	1	0	0	0	1	0	0	3	3	0	1	0	11	3^*rd*^
6	S43	0	1	0	2	0	0	1	1	2	1	1	1	1	11	3^*rd*^
7	SA2	0	2	0	0	0	3	1	0	2	1	1	0	0	10	4^*th*^
8	SA25	1	0	1	1	1	1	0	0	1	3	1	0	0	10	4^*th*^
9	S62	2	0	0	0	1	0	1	0	2	1	1	0	0	8	5^*th*^
10	SA29	2	1	1	0	1	1	0	0	1	0	1	0	0	8	5^*th*^

*Ph^*a*^, *P. hypobrunnea* (ITCC 4141); Fo^*b*^, *F. oxysporum* (MTCC 284); Pt^*c*^, *P. thaea* (ITCC 6599); Ev^*d*^, *E. vexans* (ITCC 938); Rs^*e*^, *R. solani* (MTCC 4633); Ns^*f*^, *N. sphaerica* (KJ767520); Chi^*g*^, Chitinase production; Sid^*h*^, Siderophore production; IAA^*i*^, Indole acetic acid production; PS^*j*^, Phosphate solubilization; AM^*k*^, Ammonia production; ACC^*l*^, ACC deaminase production.*

### Biochemical Profile of Top Ranked Isolates

The three highest ranked isolates based on a bonitur scale of ranking, SA1, T1LA3, and SA14 were subjected to biochemical profiling mainly to assess their carbohydrate utilization, β-galactosidase activity, esculin hydrolysis, citrate and malonate utilization consisting a total of 35 tests. Isolate SA1 showed positive for 18 tests, isolate T1LA3 showed 29 tests and isolate SA14 showed 25 positive tests (Table 5 and Supplementary Figure 2).

**TABLE 5:** Biochemical profiling of actinobacterial isolates SA1, T1LA3, and SA14 (KB009 HiCarbohydrate kit, Himedia).

**Sl. No.**	**Test**	**SA1**	**T1LA3**	**SA14**
1	Lactose	−	+	−
2	Xylose	+	+	−
3	Maltose	+	+	−
4	Fructose	+	+	+
5	Dextrose	+	+	+
6	Galactose	−	−	−
7	Raffinose	−	−	−
8	Trehalose	+	+	+
9	Melibiose	−	+	+
10	Sucrose	−	+	+
11	L-Arabinose	−	+	+
12	Mannose	−	+	−
13	Inulin	+	−	−
14	Sodium gluconate	−	−	+
15	Glycerol	+	+	+
16	Salicin	+	+	+
17	Dulcitol	+	+	+
18	Inositol	-	+	+
19	Sorbitol	-	+	+
20	Mannitol	+	+	+
21	Adonitol	-	+	+
22	Arabitol	+	+	+
23	Erythritol	+	+	+
24	α-Methyl-D-glucoside	−	−	−
25	Rhamnose	+	−	+
26	Cellobiose	+	+	+
27	Melezitose	−	+	+
28	α-Methyl-D-mannoside	−	+	+
29	Xylitol	−	+	+
30	ONPG	+	+	−
31	Esculin hydrolysis	+	+	+
32	D-Arabinose	+	+	−
33	Citrate utilization	+	+	+
34	Malonate utilization	+	+	+
35	Sorbose	−	+	+
36	Control	−	−	−

*+ color change or activity detected; − No color change or no activity detected.*

### Fungal Hyphal Morphology of *P. hypobrunnea* as Affected by Isolate T1LA3

**FIGURE 3:**
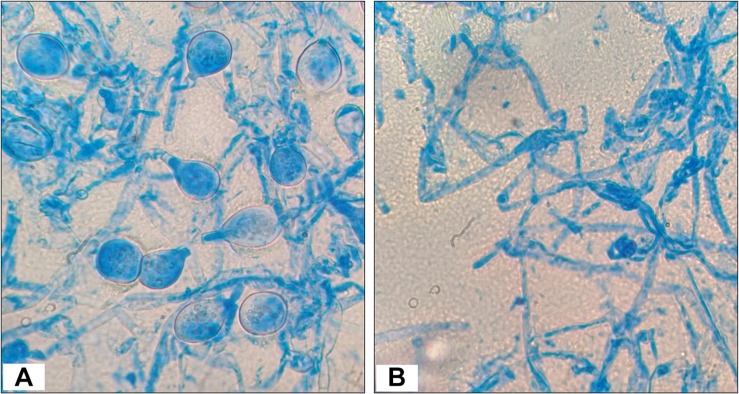
Morphology observations of *Poria hypobrunnea* (ITCC 4141) mycelia stained with lactophenol cotton blue under light microscope (100X). **(A)** Fungal mycelia from the control plate and **(B)** fungal mycelia from the zone of inhibition.

The test fungal pathogen (control sample) had a homogeneous structure and showed luxurious growth and spore formation. However, the fungal mycelia from the edge of zone of inhibition or interaction showed less extensive growth and no spore formation (Figure 3).

### Phosphate Solubilization Efficiency of the Endophytic Actinobacterial Isolates

The three isolates, SA1, SA14 and S85 were quantitatively tested using colorimetric assay for their efficiency to solubilize phosphate at different initial pH of 6.0, 7.0 and 8.0 (alkaline, neutral and acidic pH). All the isolates were able to grow in the culture medium at all of the initial pH values studied. The isolates showed higher phosphate solubilization at neutral pH of 7.0 compared to acidic (6.0) and alkaline pH (8.0) (Supplementary Table 1).

### Confirmation of IAA by TLC, HPLC, and LC-MS

**FIGURE 4:**
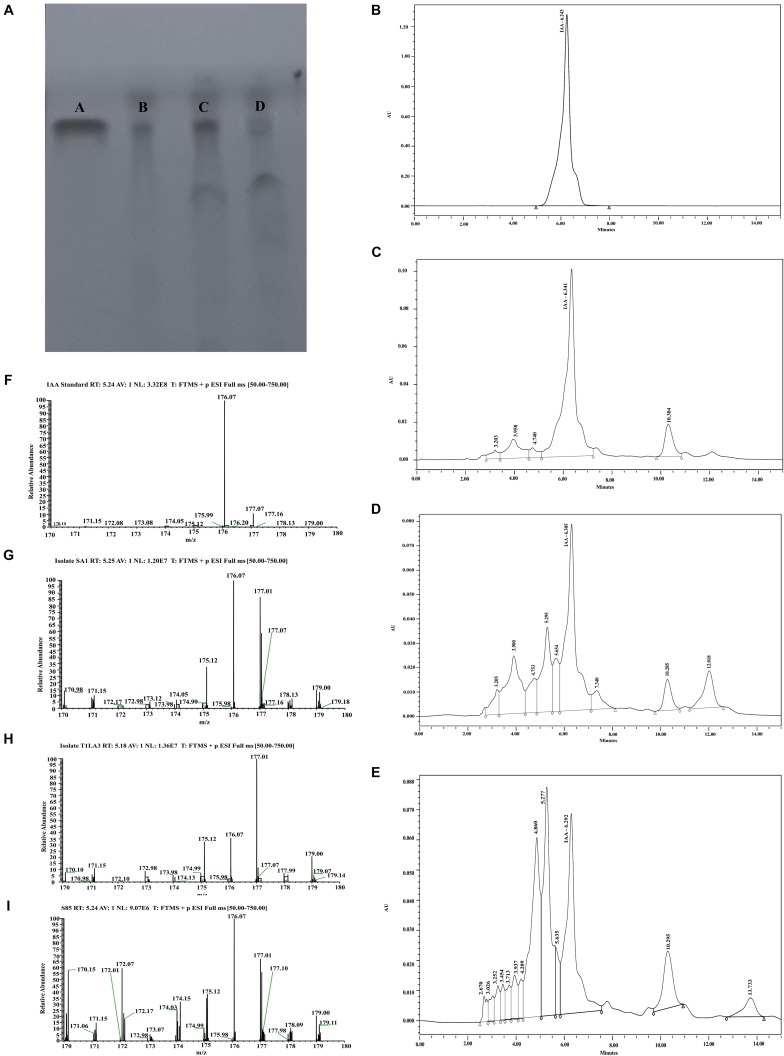
Detection orf IAA production by isolates, SA1, T1LA3, and S85 using chromatographic methods. **(A)** thin-layer chromatography (TLC) – A. standard IAA, B. SA1, C. T1LA3, and D. S85; High- performance liquid chromatography (HPLC) – **(B)** standard IAA, **(C)** SA1, **(D)** T1LA3, **(E)** S85; Liquid chromatography–mass spectrometry (LC-MS) - **(F)** standard IAA, **(G)** SA1, **(H)** T1LA3, **(I)** S85.

The production of IAA from endophytic actinobacteria isolates, SA1, T1LA3, and S85 was confirmed by TLC. As shown in Figure 4A, spots separated on TLC plates were observed under UV light at 245 nm and developed by Salkowski reagent. The results revealed that the putative IAA samples extracted from the isolates and the standard IAA showed similar retention factor (RF) value of 0.69. Similarly, the HPLC elution chromatogram of the IAA standard (Sigma) exhibited a major peak at a retention time of 6.243 min, while IAA isolated from SA1 appeared as a sharp peak at a similar retention time of 6.341 min, T1LA3 at 6.305 min, and S85 at 6.292 min (Figures 4B–E). LC-MS was used to determine more precisely the presence of IAA in the bacterial supernatant of the endophytic actinobacteria isolates SA1, T1LA3, and S85. A solution of standard IAA (0.5 mg/mL) prepared in methanol was used to determine the retention time of IAA in the LC-MS system (Figures 4F–I). Detection of IAA with positive ionization resulted in a major peak at m/z 176.07 in extracted ion chromatogram. Using the peak area at m/z 176.07 of the samples, we detected IAA in the supernatants of the isolates, SA1, T1LA3, and S85 (Figures 4F–I).

### Diversity of Culturable Endophytic Actinobacteria Associated With *Camellia* spp.

#### ARDRA (Amplified Ribosomal DNA Restriction Analysis) and BOX-PCR Fingerprinting

**FIGURE 5:**
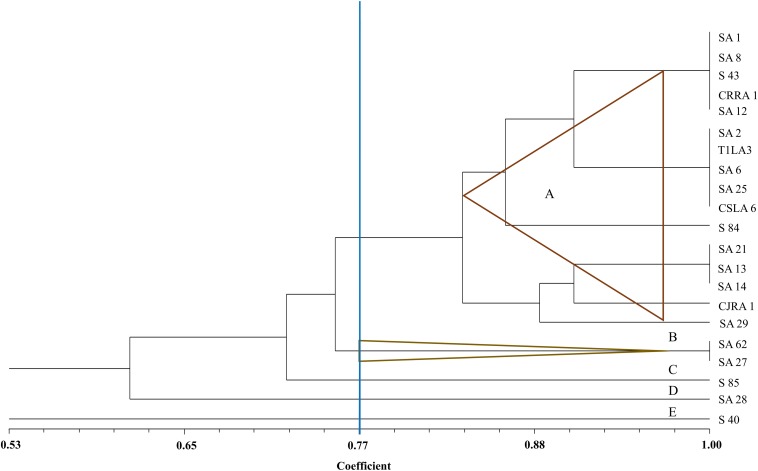
UPGMA dendrogram based on the 16S rDNA PCR-RFLP fingerprints of endophytic actinobacteria strains isolated from tea species with antifungal activity.

**FIGURE 6:**
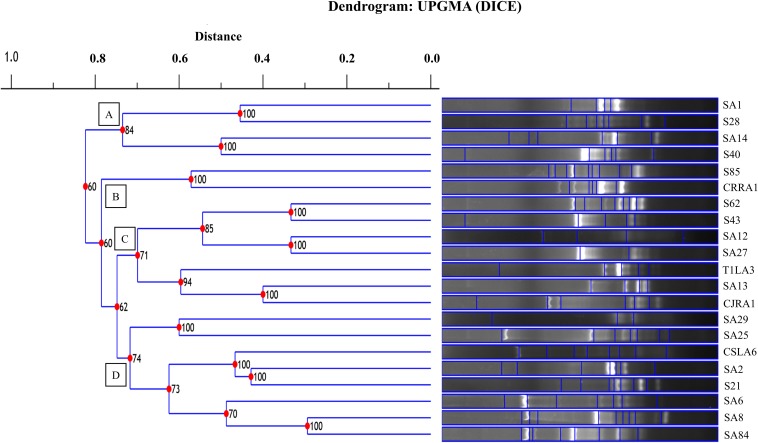
UPGMA dendrogram based on the BOX PCR fingerprints of endophytic actinobacteria strains isolated from tea species with antifungal activity.

The restriction digestion profiles of all the 21 endophytic actinobacteria with antifungal traits were analyzed and a dendrogram was generated. The 21 endophytic actinobacterial strains isolated from 6 different tea species were grouped into 5 separate clusters, A-E at 77% similarity levels in ARDRA analysis. The isolates formed one dominant group comprising 16 strains which were divided into subgroups (Figure 5). The other 4 groups contained 1-2 strains. All the *Streptomyces* spp. were clustered in group A and *Microbispora* spp. were clustered into group B on the basis of ARDRA banding patterns. The BOX-PCR fingerprinting of all the selected antagonistic endophytic actinobacteria isolates were performed and the bands of sizes between 500 bp to 5 kb were considered for the scoring to obtain a dendrogram according to UPGMA using DICE similarity coefficient. Analysis of the BOX-PCR fingerprinting showed that the 21 strains were grouped into 4 clusters, A, B, C and D each consisting of 2–8 strains (Figure 6).

#### 16S rRNA Gene Sequencing

**FIGURE 7:**
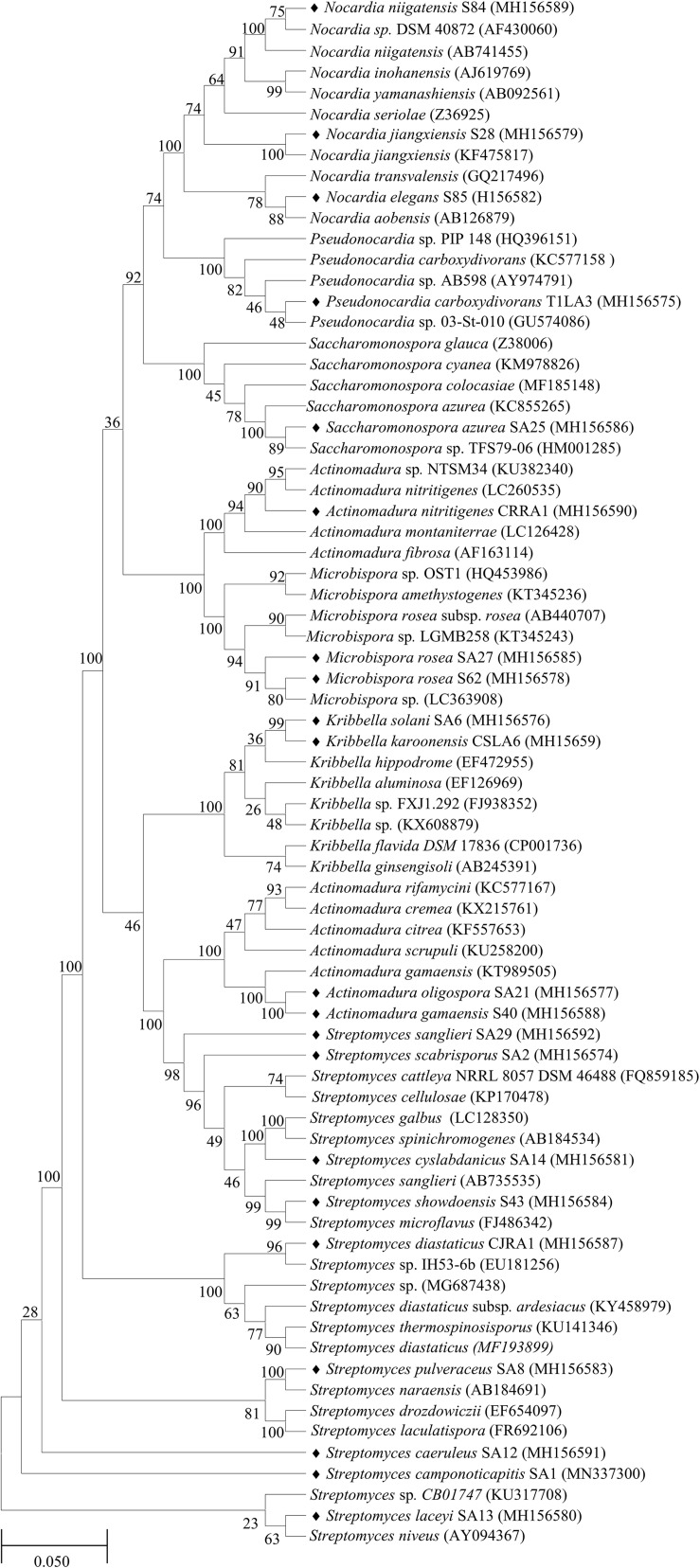
Phylogenetic tree based on NJ method of 16S rRNA gene sequences of endophytic actinobacteria with antifungal activity associated with tea species. The bootstrap consensus tree inferred from 1000 replicates is taken to and branches corresponding to partitions reproduced in less than 50% bootstrap replicates are collapsed. The percentage of replicate trees in which the associated taxa clustered together in the bootstrap test (1000 replicates) are shown next to the branches. The evolutionary distances were computed using the p-distance method and are in the units of the number of base differences per site. All positions with less than 50% site coverage were eliminated. That is, fewer than 50% alignment gaps, missing data, and ambiguous bases were allowed at any position. The scale bar represents 0.05 substitutions per nucleotide position.

The 16S rRNA gene sequences of all the endophytic actinobacteria were aligned with 98–100% similar sequence retrieved from database. The strains belong to the orders *Corynebacteriales, Pseudonocardiales*, *Streptomycetales*, *Propionibacteriales*, *Streptosporangiales*, and *Micrococcales*. The majority of the isolates, 28 out of 46 (60.8%) belong to the genus, *Streptomyces*. The strains represented 9 genera *Nocardia, Amycolatopsis, Streptomyces, Pseudonocardia, Kribbella, Actinomadura, Microbispora, Rothia* and *Saccharomonospora* (Table 6). The National Center for Biotechnology Information GenBank accession numbers for the sequences of endophytic actinobacteria are from MH156573 to MH156593, MN337298 to MN337321 and MG779620. The 16 S rRNA gene sequence of the 21 potent antifungal actinobacteria were aligned with similar sequence to obtain a phylogenetic tree based on NJ method (Figure 7).

**TABLE 6:** Endophytic actinobacteria isolated from *Camellia* spp., and the closest type strains based on 16S rRNA gene sequence similarity.

**Sl. No.**	**Isolate code**	**Accession no.**	**Base pair**	**Closest hit taxon**	**Similarity (%)**
1	SA1	MN337300	1,405 bp	*Streptomyces niveus* NRRL 2466	99.64
2	SA2	MH156574	1,405 bp	*Streptomyces hyalinus* MB891-A1(T)	99.64
3	T1LA3	MH156575	1,371 bp	*Pseudonocardia carboxydivorans* Y8	99.71
4	SA16	MN337298	1,396 bp	*Streptomyces camponoticapitis* 2H-TWYE14(T)	99.57
5	SA6	MH156576	1,404 bp	*Kribbella hippodromi* S1.4	98.99
6	CSLA2	MN337299	1,402 bp	*Streptomyces niveus* NRRL 2466(T)	99.64
7	CJRA1	MH156587	1,400 bp	*Streptomyces diastaticus subsp. Ardesiacus* NRRL B-1773	100
8	S40	MH156588	1,397 bp	*Actinomadura rhizosphaerae* SDA37	98.84
9	SA21	MH156577	1,404 bp	*Actinomadura logoneensis* NEAU-G17(T)	98.57
10	SA5	MN337302	1,399 bp	*Streptomyces sanglieri* NBRC 100784	99.57
11	CSLA5	MN337303	1,395 bp	*Streptomyces niveus* NRRL 2466	99.64
12	S51	MN337304	1,406 bp	*Streptomyces cyslabdanicus* K04-0144	99.22
13	S62	MH156578	1,399 bp	*Microbispora rosea* ATCC 12950	99.64
14	S28	MH156579	1,394 bp	*Nocardia jiangxiensis* NBRC 101359	98.78
15	T1LA5	MN337317	1,359 bp	*Streptomyces yanii* NBRC 14669	98.80
16	S39	MN337305	1,396 bp	*Nocardia jiangxiensis* NBRC 101359	99.00
17	SA13	MH156580	1,405 bp	*Streptomyces laceyi* NRRL 2466	99.64
18	SA14	MH156581	1,407 bp	*Streptomyces cyslabdanicus* K04-0144	99.22
19	SA9	MN337316	1,406 bp	*Streptomyces corchorusii* DSM 40340(T)	98.79
20	S41	MN337313	1,397 bp	*Streptomyces niveus* NRRL 2466	99.64
21	S85	MH156582	1,396 bp	*Nocardia africana* DSM 44491	99.36
22	S42	MN337307	1,388 bp	*Streptomyces niveus* NRRL 2466 (T)	99.57
23	SA10	MN337320	1,394 bp	*Streptomyces kebangsaanensis* SUK12	99.57
24	SA20	MN337321	1,416 bp	*Rothia koreensis* P31	95.39
25	S84	MH156589	1,392 bp	*Nocardia niigatensis* NBRC 100131	98.99
26	CJRA5	MN337308	1,389 bp	*Nocardia Africana* DSM 44491	99.57
27	CJRA2	MN337309	1,399 bp	*Nocardia Africana* DSM 44491	91.97
28	CSLA7	MN337310	1,402 bp	*Streptomyces niveus* NRRL 2466(T)	99.71
29	CRRA1	MH156590	1,394 bp	*Actinomadura nitritigenes* DSM 44137	99.57
30	SA7	MN337311	1,379 bp	*Streptomyces hyalinus* MB891-A1(T)	99.78
31	SA8	MH156583	1,404 bp	*Streptomyces pulveraceus* LMG 20322	99.57
32	SA4	MN337312	1,390 bp	*Streptomyces niveus* NRRL 2466(T)	99.78
33	S43	MH156584	1,405 bp	*Streptomyces sanglieri* NBRC 100784	99.72
34	S72	MN337315	1,405 bp	*Streptomyces cyslabdanicus* K04-0144	99.29
35	CSR1	MN337318	1,397 bp	*Streptomyces sanglieri* NBRC 100784	99.64
36	SA27	MH156585	1,395 bp	*Microbispora rosea* ATCC 12950	99.78
37	SA35	MN337319	1,399 bp	*Streptomyces hyalinus* MB891-A1(T)	99.79
38	SA17	MG779620	1,399 bp	*Streptomyces kebangsaanensis* SUK12	98.00
39	SA12	MH156591	1,403 bp	*Streptomyces camponoticapitis* 2H-TWYE14	99.43
40	SA29	MH156592	1,402 bp	*Streptomyces yanii* NBRC 14669	95.26
41	SA3	MH156573	1,384 bp	*Amycolatopsis pretoriensis* DSM 44654	88.79
42	SA25	MH156586	1,414 bp	*Saccharomonospora azurea* NA-128	99.15
43	CSR3	MN337306	1,403 bp	*Streptomyces niveus* NRRL 2466	99.71
44	CSLA6	MH156593	1,434 bp	*Kribbella hippodromi* S1.4	*98.92*
45	CSR4	MN337314	1,395 bp	*Streptomyces hyalinus* MB891-A1(T)	99.57
46	SA11	MN337301	1,412 bp	*Kribbella pittospori* PIP 158	98.59

#### Molecular Identification and Detection of Biosynthetic Genes

Molecular detection of biosynthetic genes revealed that 12 strains out of the 21 potent strains were positive for chitinase 18 Glycosyl Hydrolase family gene. All the isolates showing positive chitinase activity in plate assay were found to be positive for the presence of chitinase 18 Glycosyl Hydrolase family gene. *Streptomyces* was found to be the major chitinase producing genera with six isolates showing positive for chitinase gene. Also, 11 isolates showed the presence of NRPS gene and one was positive for PKS-1 gene. Strain SA8 *Streptomyces* sp. is positive for all the biosynthetic genes tested, i.e. chitinase, NRPS and PKS (Supplementary Figure 3).

#### Tea Plant Growth Promotion by Selected Actinobacterial Isolates

**FIGURE 8:**
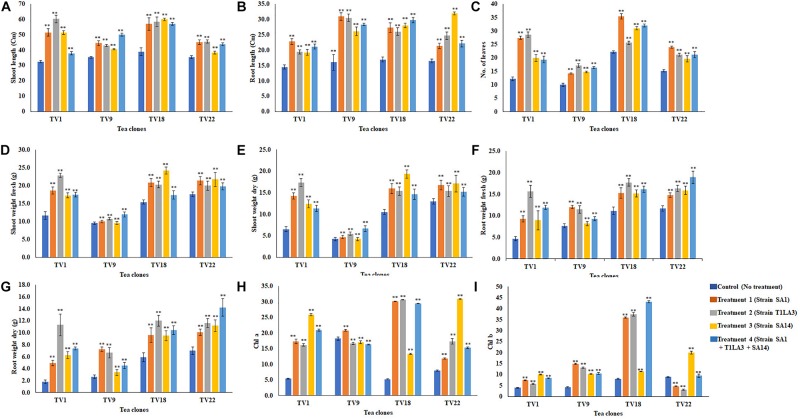
*In vivo* plant growth promoting effect of endophytic actinobacterial isolates on different commercial clones (TV1, TV9, TV18 and TV22) of tea. **(A)** Shoot length (cm), **(B)** root length (cm), **(C)** No. of leaves, **(D)** Shoot weight fresh (g), **(E)** Shoot weight dry (g), **(F)** Root weight fresh (g), **(G)** Root weight dry (g), **(H)** Chlorophyll a (mg/g fresh weight), **(I)** Chlorophyll b (mg/g fresh weight). The values are mean ± Standard error (*n* = 3). ^∗∗^Represents statistical significance (*p* < 0.05, Two-way ANOVA).

**FIGURE 9:**
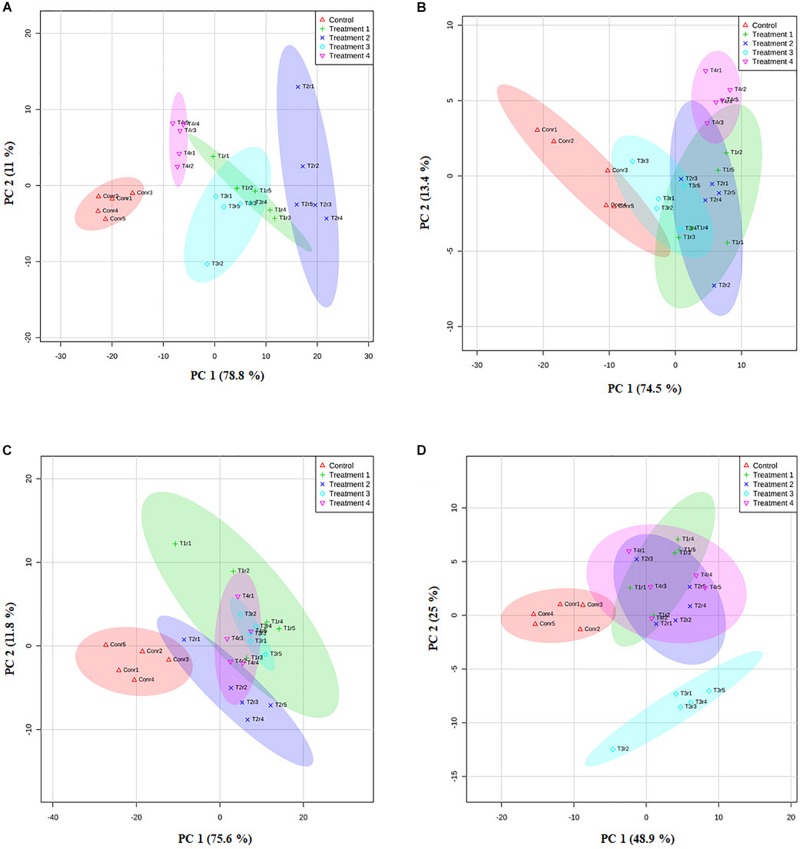
Principle component analysis based on the eigen values in a matrix on the PGP datasets to study the correlation between growth parameters and bacterial treatment in the PGP experiment. **(A)** TV1, **(B)** TV9, **(C)** TV18, and **(D)** TV22.

We further analyzed the efficacy of potent endophytic actinobacterial isolates for PGP in treated plants compared to untreated control plants (Supplementary Figures 4a,b). All the endophytic isolates showed significant differences (*P* ≤ 0.05) in terms of plant growth promoting parameters in the treated plants compared to untreated control. The growth parameters including fresh and dry weight were significantly increased in the treated tea clones (Figure 8). The maximum increase was observed in root dry weight of TV1 clone with treatment 2 (T1LA3) and consortia by 6.2- and 4.05-fold, respectively. However, the degree of PGP in terms of no. of leaves (1.15 to 2.3 fold), shoot dry weight (1.16 to 2.6 fold), root fresh weight (1.0 to 3.3 fold), shoot fresh weight (1.12 to 1.9 fold), shoot height (1.0 to 1.8 fold), root length (1.2 to 1.9 fold) varied with various endophytic actinobacteria treatments in different tea clones (Supplementary Figures 5a–p). The enhancement in the chlorophyll content in actinobacteria treated tea clones signifies their effect on photosynthetic activity and growth of the plant (Figure 8). PCA reduced the various PGP parameters to two principle components, PC1 and PC2 in which PC1 represents the maximum variance in the dataset followed by PC2. More than 75% of the variation are represented by PC1 in tea clones TV1, TV9 and TV18 and 48% in TV22. The treatments formed separate groups in all the tea clones showing variance between treated and control groups (Figure 9). In addition, a significant difference in the chlorophyll a and b content of the treated tea clones was observed compared to the control group (*P* ≤ 0.05). Soil parameter analysis showed considerable differences in total phosphorus content in TV1 and TV9 tea clones especially when treated with strain SA1. Calcium was not detected in any of the soil samples (Supplementary Tables 2a–d).

## Discussion

Tea is widely used as a beverage due to its medicinal value and caffeine content and has a high economic value (Gebrewold, 2018). Like numerous plant species, tea plants host endophytic actinobacteria that are unexplored for microbial natural product discovery. Many previous studies of different host plants have indicated the presence of diverse actinobacteria in plants, including tomato (Fialho de Oliveira et al., 2010), Chinese licorice (Zhao et al., 2018). However, the diversity and function of endophytic actinobacteria in wild *Camellia* spp. and related genera are not well studied.

Since the endophytic community varies from genera to genera and species to species, sampling plants from different species and genera increases the possibility to isolate different endophytic actinobacteria with varied PGP characteristics. We isolated actinobacteria from the leaves and roots of different *Camellia* spp. such as *C. sinensis*, *C. japonica*, *C. sasanqua*, *C. rosiflora*, and *C. drupifera*, and related genera, *Eurya japonica* and tested for PGP and antifungal traits. In this study, we isolated nine genera of actinobacteria including *Streptomyces* and other rare actinobacteria like *Nocardia, Amycolatopsis, Pseudonocardia, Kribbella, Actinomadura, Microbispora, Rothia* and *Saccharomonospora*. Most of them have been isolated previously and reported as endophytes from various crops (Hasegawa et al., 2006; Shimizu, 2011; Matsumoto and Takahashi, 2017).

The endophytic actinobacteria associated with plant tissues are capable of promoting plant growth by making nutrients/substrates (e.g., phosphorous, nitrogen and iron) accessible to the host plant, producing various plant hormones and protection from phytopathogens (Liu et al., 2017). In this study, we evaluated different PGP characteristics such as IAA production, phosphate solubilization, ammonia production, siderophore production. Out of 46 endophytic actinobacterial strains associated with *Camellia* spp., 37 (80.4%) strains showed at least one PGP or biocontrol trait. Plant growth can be enhanced by the microbial production of phytohormones like IAA which stimulates root elongation and induces the formation of adventitious roots (Sousa and Olivares, 2016). As in previous studies, both *Streptomyces* and non-*Streptomyces* spp. were reported as IAA producers and within a similar range (Nimnoi et al., 2010; Shutsrirung et al., 2013; Singh and Dubey, 2018). Phosphorus is a major micronutrient responsible for many functional processes and phosphate solubilizing bacteria (PSB) can increase the availability of phosphorus for the plants. PSB can also increase the accessibility of phosphate in soil by producing hydrolyzing enzymes like acid phosphatase that can hydrolyze inorganic phosphate. As previously reported, a common feature of endophytic actinobacteria is their potential to solubilize inorganic phosphate (Afzal et al., 2019). In the present study, the percentage of total phosphate solubilizers is within the range of previously reported phosphate solubilizers (Qin et al., 2011; Passari et al., 2015), including 14 *Streptomyces* strains. Biological nitrogen fixation is the mechanism that converts atmospheric nitrogen (N_2_) to ammonia NH_3_ or NH_4_^+^ which can be easily used by plants and is carried out by various groups of microbial population (Meena et al., 2017). Nitrogen fixing and ammonia producing bacteria are effective in N_2_ assimilation, and have been used as inoculants for growth promotion and increasing yield of crops (Marques et al., 2010). Many *Streptomyces* spp. as well as rare actinobacteria are known to produce ammonia (Nimnoi et al., 2010) and aid in plant growth promotion as reported in this study. Ammonia production is an indirect method of PGP and also plays an important role in suppressing the growth of fungal plant pathogens (Minaxi et al., 2012). Another important trait of the endophytic actinobacteria that influences their PGP and antagonistic activities is the production of iron sequestering molecules, siderophores. In the present investigation only three isolates were able to produce siderophores. Similar results were obtained in *Jatropha* (Qin et al., 2011). Also, about 28.2% of the total endophytic actinobacterial isolates were positive ACC deaminase producers. ACC deaminase plays a significant role in the reduction of stress ethylene in host plants, thus aiding in withstanding abiotic stress, especially drought (Kang et al., 2010).

Antagonistic microbes that produced hydrolytic enzymes such as cellulase, protease, pectinase and chitinase are capable of degrading fungal and bacterial cell wall, cell membrane, cell membrane proteins, and extracellular virulence factors have been implicated in biocontrol of plant diseases (Palaniyandi et al., 2013). In addition, the production of cell-wall hydrolyzing enzymes, mainly pectinases and cellulases are involved in bacterial entrance and spreading within the plant (Afzal et al., 2019). Most of the isolates screened in this study produced at least one of the extracellular enzymes. Endophytic actinobacteria associated with plants have the ability to minimize the challenges imposed by phytopathogens including the genus, *Streptomyces*, which is widely recognized for their ability to synthesize numerous bioactive metabolites that play an important role in the phytopathogen control (Sousa and Olivares, 2016; Dinesh et al., 2017). In the present study, *Streptomyces* and non-*Streptomyces* species, both showed potent antifungal activity against at least one of the tested fungal phytopathogens. Two isolates SA25 *Saccharomonospora* sp. and SA29 *Streptomyces* sp. showed broad spectrum antifungal activity against all the tested pathogens. However, the highest percentage of inhibition was shown by SA21 *Actinomadura* sp. against *P. theae* followed by SA1 *Streptomyces* sp. against *R. solani* and T1LA3 *Pseudonocardia* sp. against *P. hypobrunnea.* In previous studies, many *Streptomyces* sp. were reported for the synthesis of antifungal metabolites as well as some reports on rare actinobacteria for antimicrobial metabolite production are reported (El-Tarabily et al., 2010; Palaniyandi et al., 2013; Singh and Dubey, 2018). Endophytic actinobacteria with positive chitinase, NRPS, PKS-1 gene showed antagonistic activity and protected plants against plant phytopathogens (Passari et al., 2016b). Most of the endophytic actinobacteria isolates having antifungal activity were positive for the presence of chitinase, NRPS or PKS-1 gene, suggesting the presence of distinctive mechanisms to inhibit the growth of pathogenic plant fungi. Four isolates were negative for any biosynthetic gene tested in this study which suggests that they might have alternative mechanisms for the inhibition of plant fungal phytopathogens.

In this study, we used the PCR fingerprinting methods, ARDRA and BOX-PCR to document the genetic diversity of the endophytic actinobacterial isolates showing potent antagonistic activity. These 21 isolates were clustered into various groups or clusters. 16S rRNA gene sequencing and BLAST analysis of the total endophytic actinobacterial isolates revealed that the isolates belonged to 9 different genera. Out of the 46 isolates, 28 (60.8%) belonged to genus *Streptomyces*, 6 (13.0%) belonged to *Nocardia*, 3 (6.5%) belonged to *Kribbella* and *Actinomadura* each, 2 (4.3%) belonged to *Microbispora*, and 1 (2.1%) belonged to *Amycolatopsis*, *Pseudonocardia*, *Saccharomonospora* and *Rothia* each. *Streptomyces* is the major genus obtained among all the actinobacterial genera. *Streptomyces* have previously been reported as the most abundant endophytic actinobacterial genera in plants such as *Jatropha*, wheat, and liquorice (Coombs and Franco, 2003; Qin et al., 2015; Zhao et al., 2018).

Bonitur assessment of the endophytic actinobacteria strains based on their PGP and biocontrol traits revealed SA1, T1LA3, SA14, and S85 as the top ranked isolates. These isolates were further tested for their ability to utilize a wide range of substrate based on the principle of pH change and substrate utilization. All three isolates were able to utilize the substrates, fructose, dextrose, trehalose, glycerol, dulcitol, salicin, mannitol, arabitol, erythritol, cellobiose, esculin, citrate, and malonate. The test fungal pathogen, *P. hypobrunnea* had a homogeneous structure and showed luxurious growth and spore formation compared to the fungal mycelia taken from the edge of the zone of inhibition. Phosphate solubilization efficiency of the isolates in different initial pH of the broth medium showed that the isolates showed highest phosphate solubilization at neutral pH by all the three isolates. In our study, the isolates retained their phosphate solubilizing activity at both alkaline and acidic pH. These findings were similar to those reported by Jena and Rath (2013), who reported that the phosphate solubilizing index of the bacterial isolates studied at different pH (pH 4-10), maximum solubilizing index (SI) was observed at neutral pH (pH 7.0) (Jena and Rath, 2013). Also, isolates SA14 and S85 showed higher phosphate solubilization at alkaline pH compared to acidic pH. Confirmation of IAA production by isolates, SA1, T1LA3 and S85 were done using chromatographic methods. TLC analysis of the ethyl acetate extracts of the samples and standard IAA showed similar RF values, consistent with previous reports (Myo et al., 2019). Furthermore, HPLC analysis of the ethyl acetate extract of the samples showed peaks at similar retention times to those of standard IAA confirming that the isolates were indeed IAA producers. Also, LC-MS analysis of the ethyl acetate extracts with positive ionization resulted in a major peak at m/z 176.07 in the chromatogram. Using the peak area at m/z 176.07 of the samples, IAA was detected in the supernatants of the tested isolates.

Endophytic actinobacterial strains with PGP traits are proposed to facilitate growth in host plants through various direct and indirect mechanisms (Afzal et al., 2019). We evaluated the efficacy of three endophytic actinobacterial strains on the growth of *C. sinensis* in a nursery experiment. The strains were selected based on ranking by using a bonitur scale of 29 points. The PGP experiment in this study was performed in commercial nursery condition using non-sterile soil because tea growing soil microbial community is diverse and complex and varies in composition, which represents a real challenge in tea cultivation. The natural microflora associated with the soil are not eliminated in order to test the effect of selected indigenous microbial inoculum compared to the untreated control carried out in 5 replicates in the same condition. The growth of all the inoculated plants in terms of different plant parameters such as fresh and dry biomass was significantly better than the uninoculated control plants. The growth of tea plants *in vivo* was not directly related to IAA production or phosphate solubilization. Treatment 2 (T1LA3) that produced IAA but didn’t solubilize phosphate performed better in TV1 and TV 18 clones, but didn’t in TV9 and TV22 clones suggesting that the performance of the PGP strains was possibly dependent on the host plant. The PGP ability of the endophytic bacteria can be shaped by the genotype of the host so that plant colonization and growth promotion ability of the endophytic bacteria seems to be a dynamic process that is structured by the genetic factors of both partners (Afzal et al., 2019).

## Conclusion

In conclusion, the endophytic actinobacteria isolated from *Camellia* spp. and related genera, *Eurya*, are represented nine different genera of actinobacteria. Most of the strains showed PGP characteristics *in vitro*, extracellular enzyme production and biocontrol trait against tea phytopathogens. The three strains selected for testing their efficacy *in vivo* were able to enhance the growth of different tea clones in nursery conditions. These strains were able to utilize a wide range of substrates. The actinobacteria inoculums utilized in this nursery experiment were assumed to have PGP effect, colonization and competent with the other native microbial population. However, interaction of these PGP strains with other native soil microflora and their colonization has to be evaluated in future to establish these strains as biofertilizers. Thus, the endophytic actinobacteria comprising PGP and biocontrol traits which may be deemed potential candidates for sustainable crop production.

## Data Availability Statement

The datasets generated for this study can be found in the GenBank-https://www.ncbi.nlm.nih.gov/nuccore/MG779620, https://www.ncbi.nlm.nih.gov/nuccore/?term=MN337298:MN337321[accn], and https://www.ncbi.nlm.nih.gov/nuccore/?term=MH156573:MH156593[accn].

## Author Contributions

DT planned, designed and supervised the research work and guided the experiments. AB conducted the laboratory and field experiments, acquired and analyzed the data and interpreted the results. AB and DT drafted the manuscript.

## Conflict of Interest

The authors declare that the research was conducted in the absence of any commercial or financial relationships that could be construed as a potential conflict of interest.
