# Supplementary material for: Phylogenetic and Functional Characterization of Culturable Endophytic Actinobacteria Associated With Camellia spp. for Growth Promotion in Commercial Tea Cultivars
Source: Front Microbiol. 2020 Feb 28;11:318. doi: 10.3389/fmicb.2020.00318 (PMC7059647; doi:10.3389/fmicb.2020.00318)
Supplement: Supplementary file 1 [file Data_Sheet_1.docx]

**Supplementary materials**

**Supplementary figure 1|** The macroscopic and microscopic characterization of selected actinobacteria strains.

**Strain T1LA3 Strain S51 Strain S62**


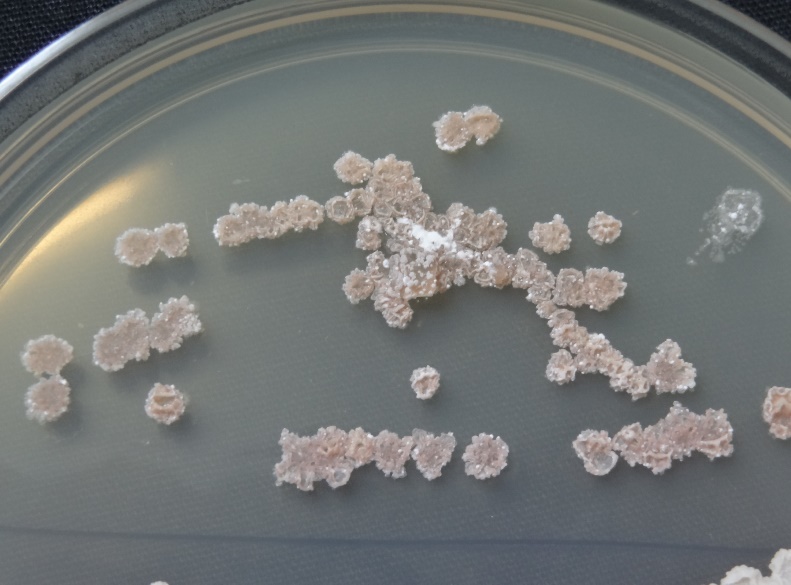

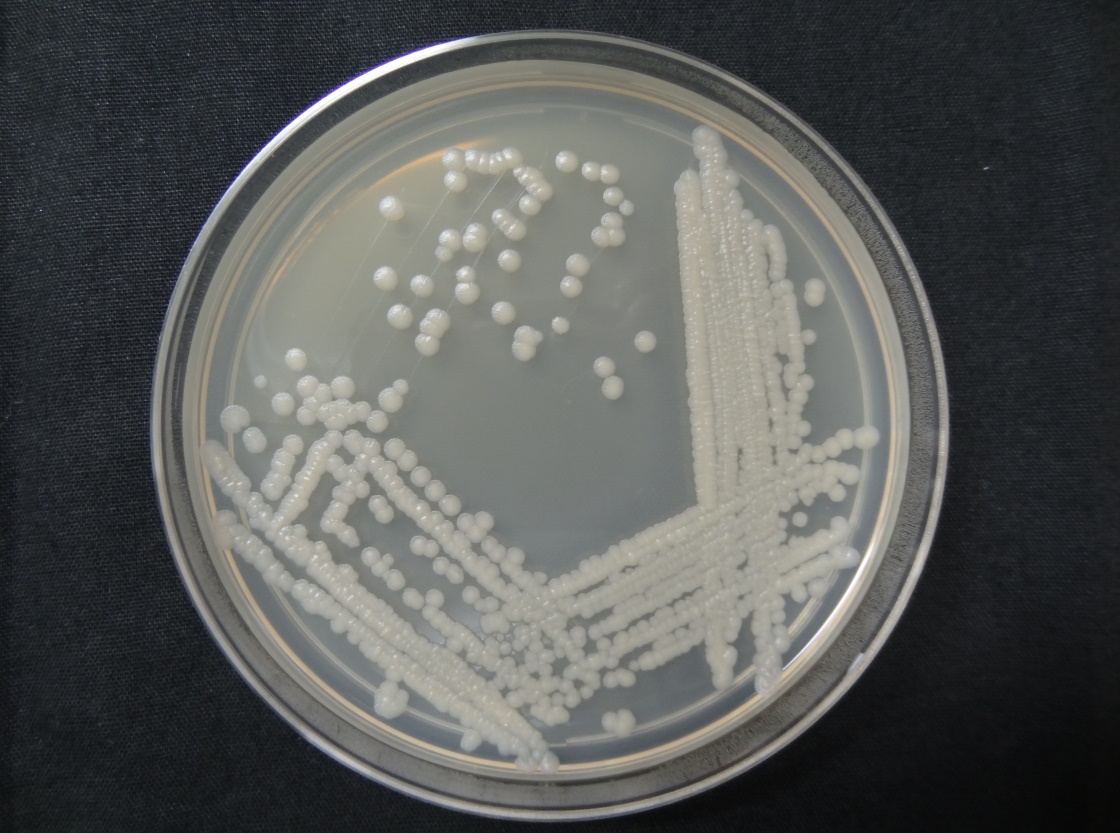

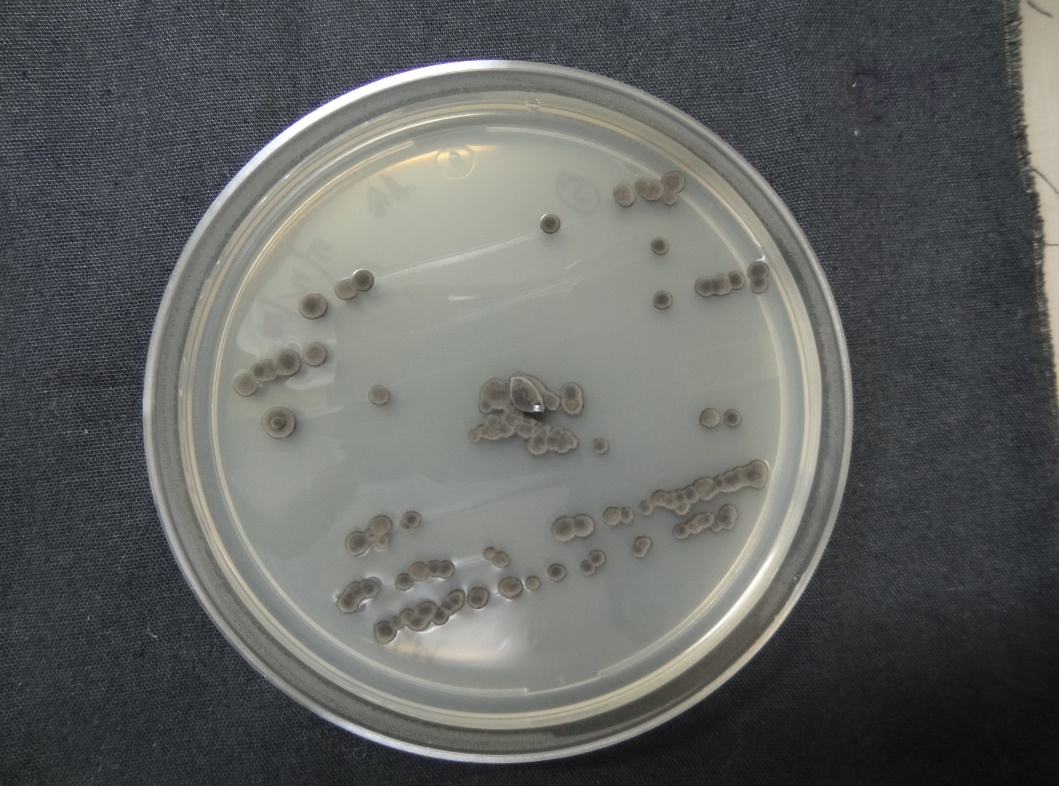


**Gram Stain GLM agar**


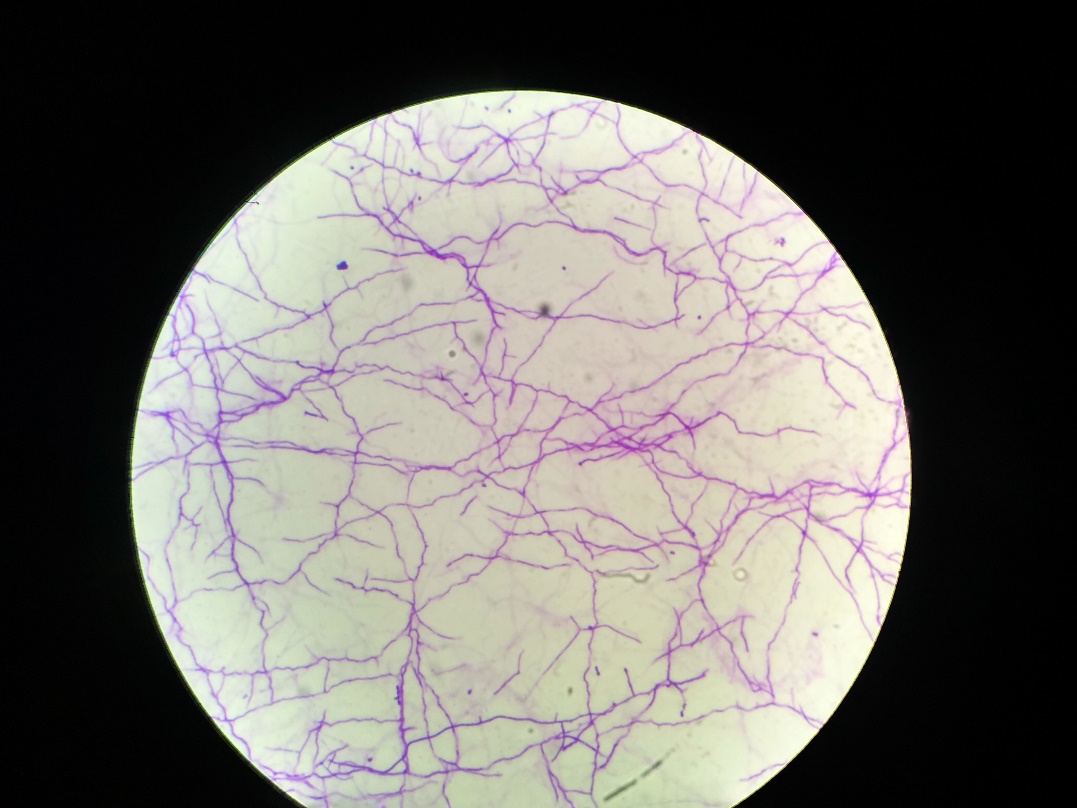

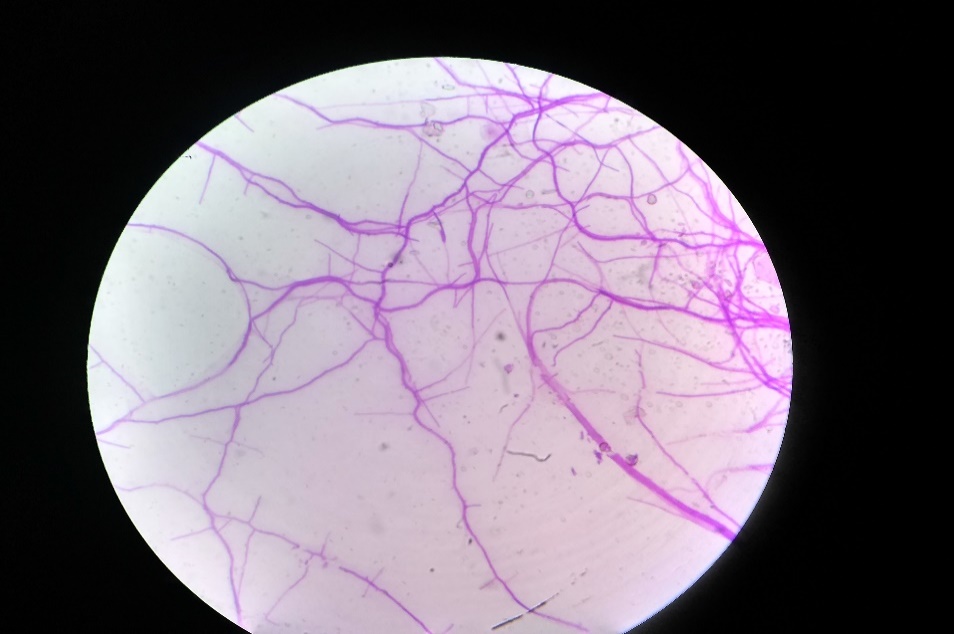

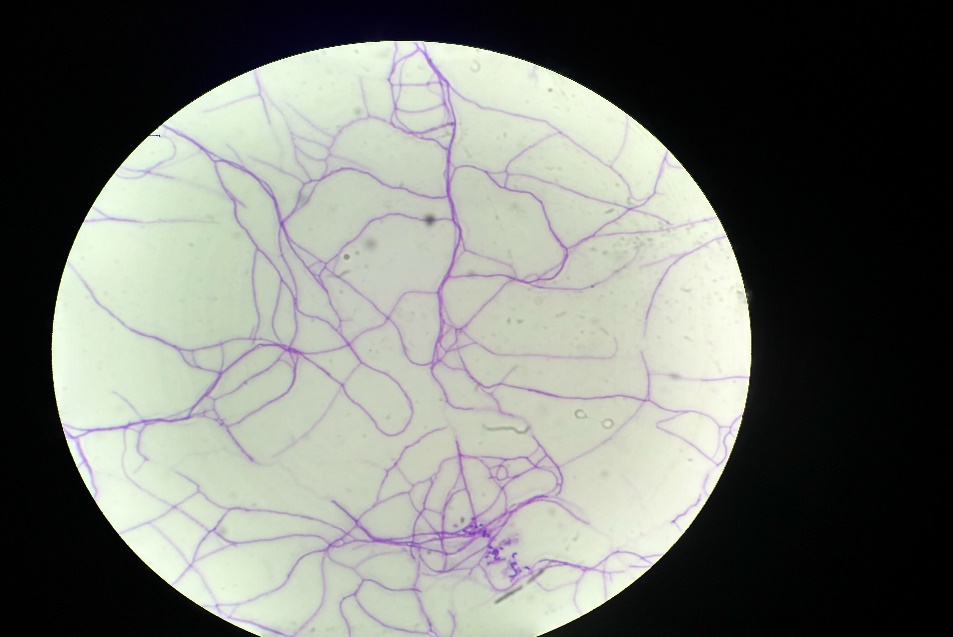


20 *μ*m

20 *μ*m

20 *μ*m

**Supplementary figure 2|** Plant growth promoting characterization of endophytic actinobacterial strains associated with tea.


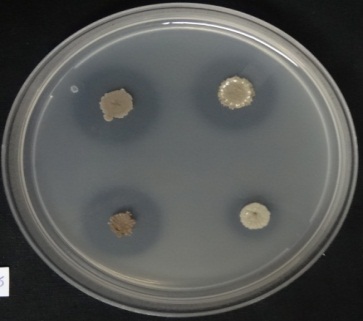

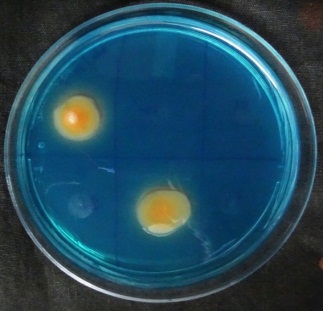

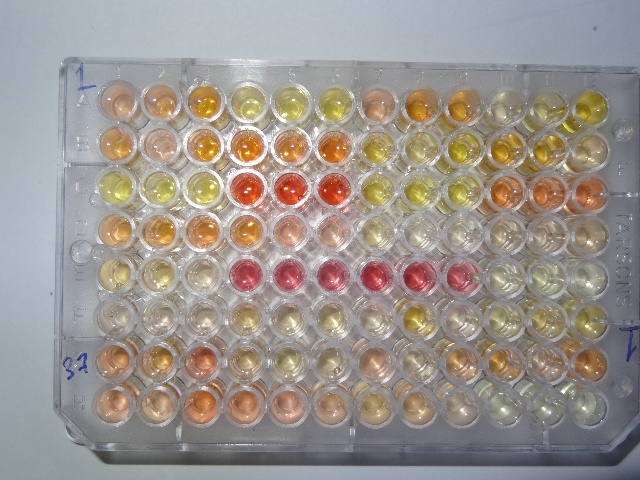


**IAA production**


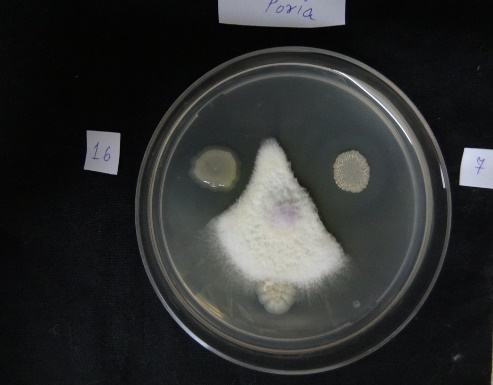


**Phosphate solubilization**

**Siderophore production**

**Antifungal activity**

**HiCarbohydrate test production**


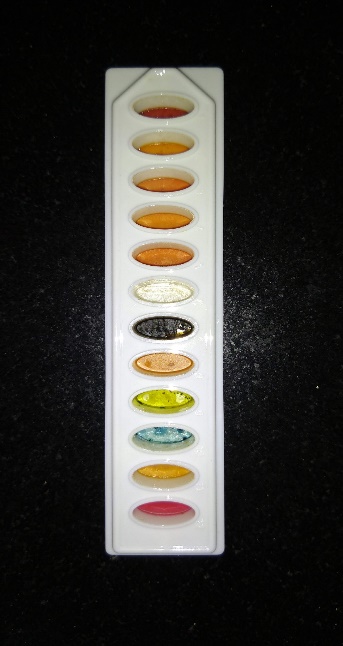

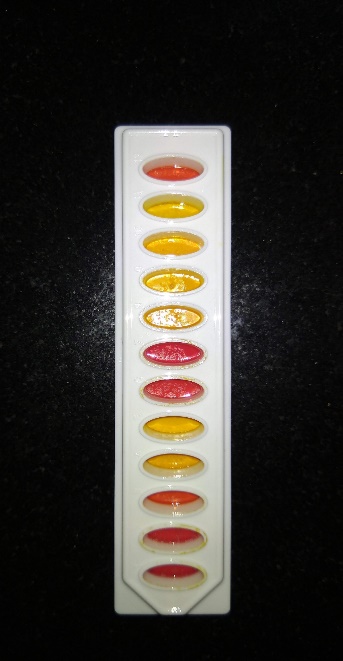

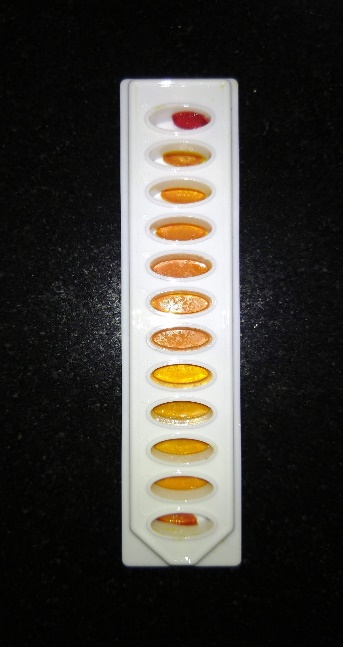


**Supplementary figure 3|** (a) 18 family chitinase gene amplification (size: 400-500 bp) with GA1F/GA1R, (b) PKS-I gene amplification (size: 1200-1400 bp) with K1F/M6R, (c) NRPS amplification (size: 700 - 800 bp) with A3F/A7R**.**


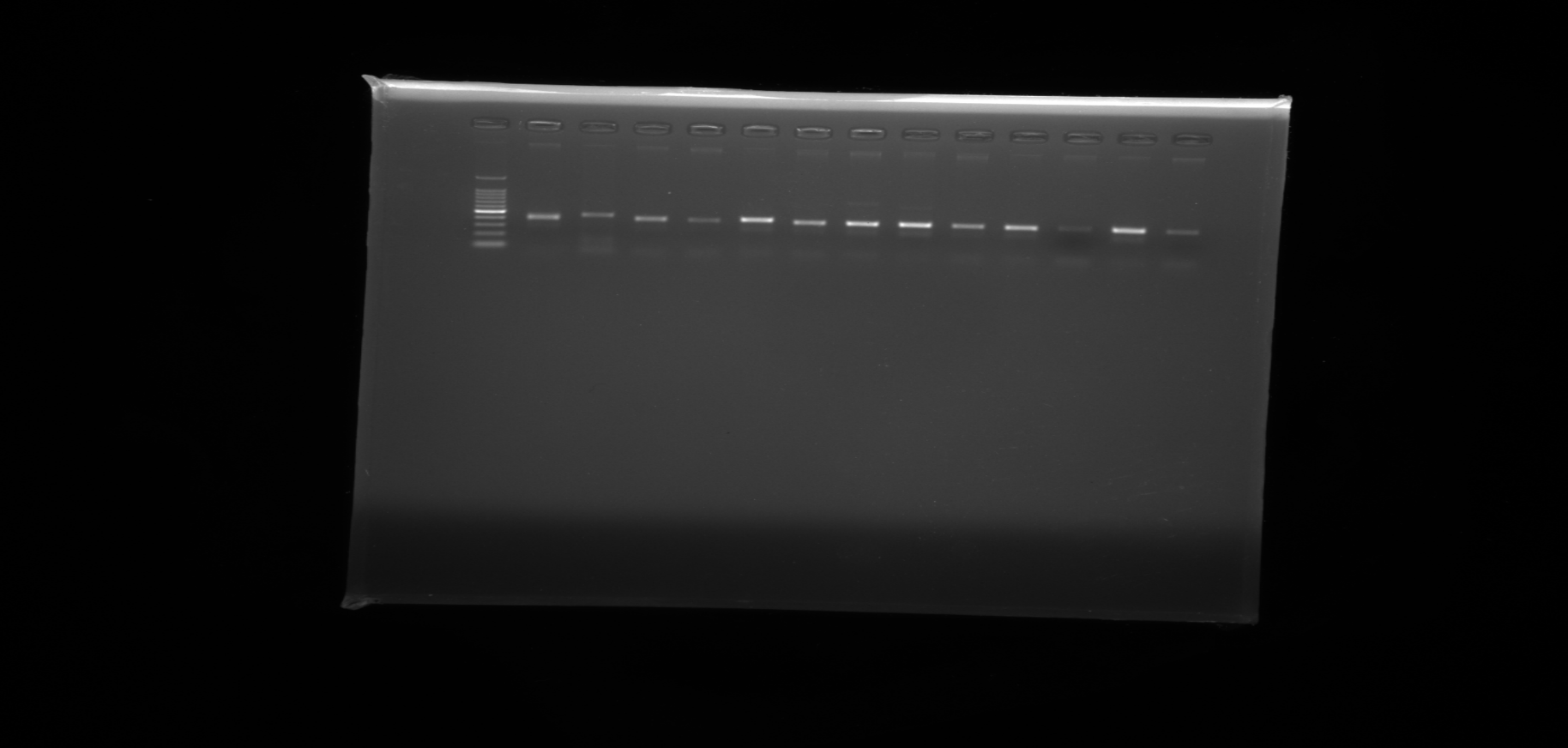


**100 bp SA1 SA2 CJRA1 SA21 S62 SA13 CRRA1 SA8 S43 SA27 SA12**


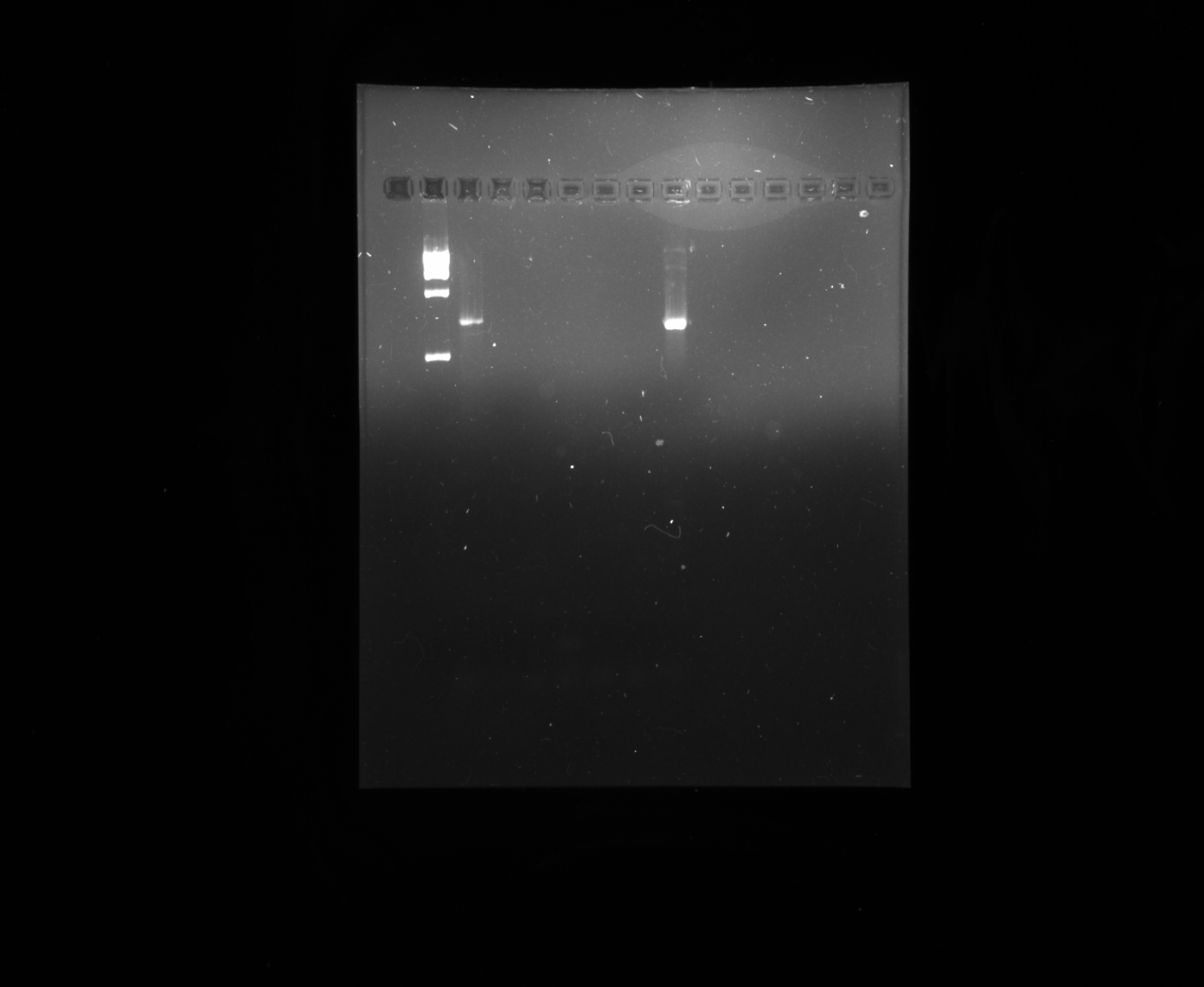


**1 kb ladder SA8**


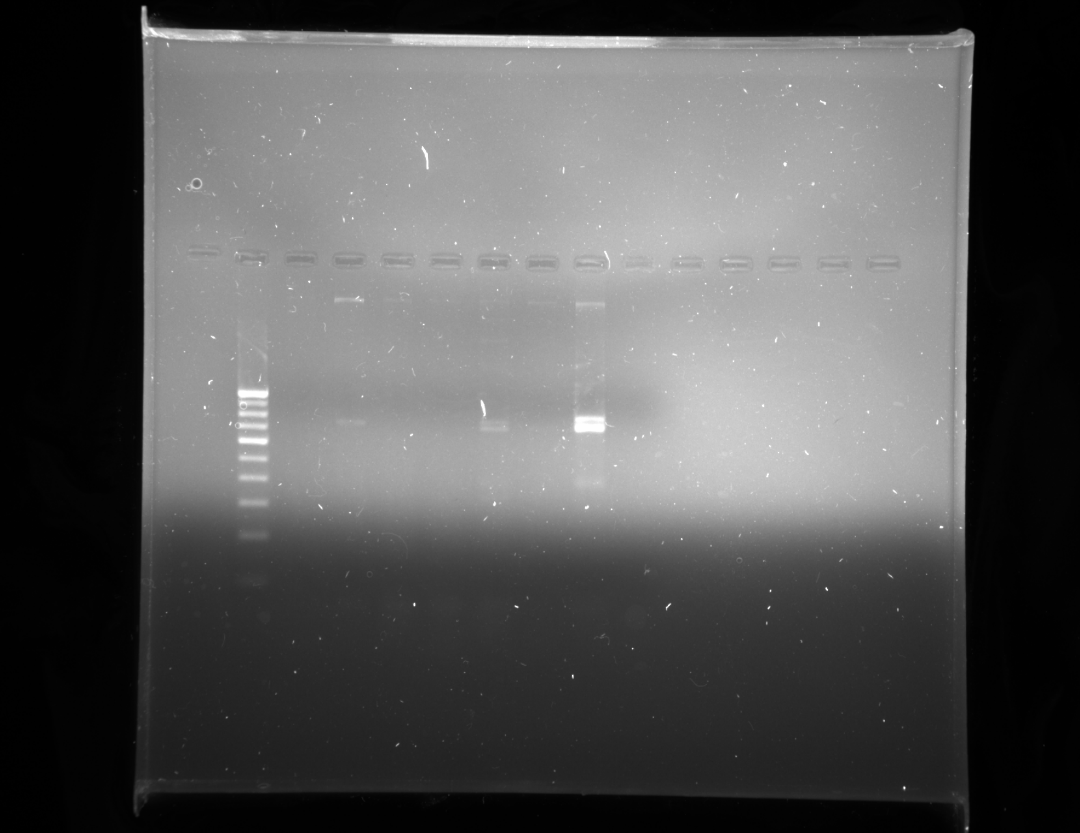


**100 bp ladder SA8**

**(a)**

**(c)**

**(b)**

**500 bp**

**700 bp**

**2000 bp**

**1000 bp**

**Supplementary figure 4|** *In vivo* plant growth promotion experiment. (a) Nursery plant growth promotion experiment, (b) Inoculated tea plant (Clone TV1) with strain T1LA3 vs control.

**
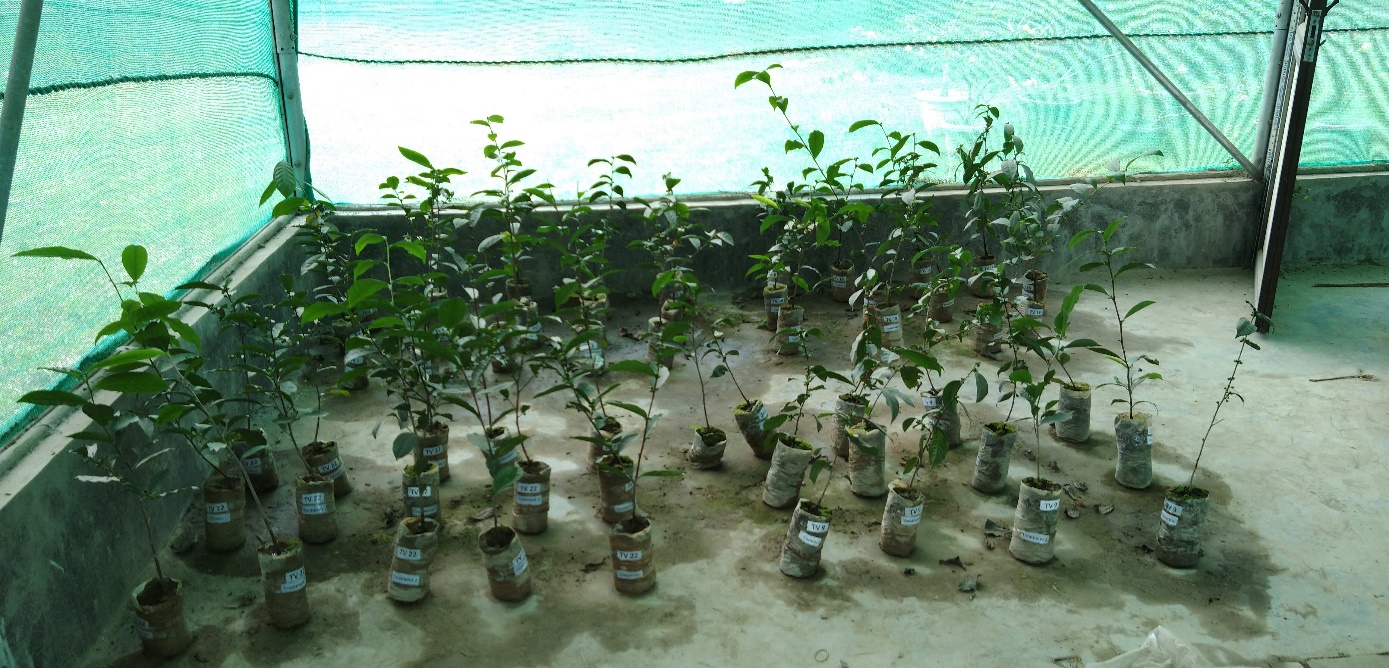
**

**(b)**


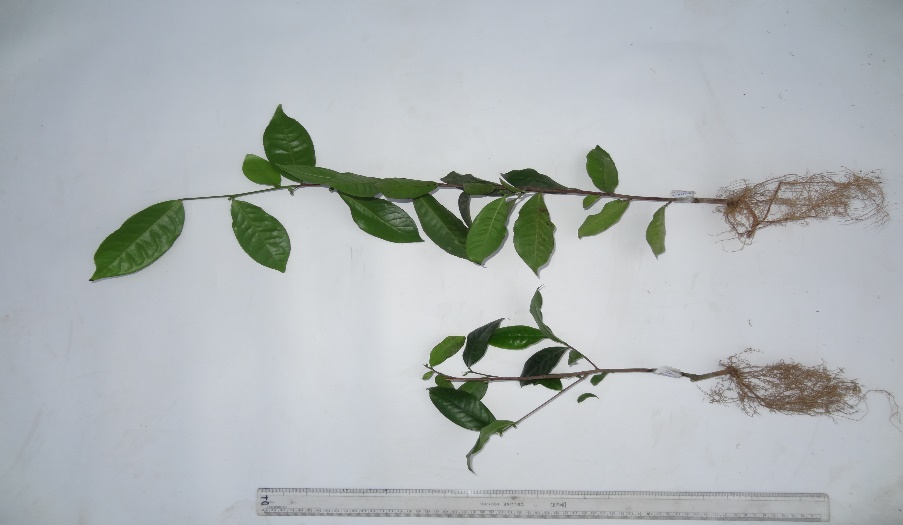


**Control**

**Treated**

**(a)**

**Supplementary figure 5|** Fold change analysis of tea clones TV1, TV9, TV18 and TV22 treated with endophytic actinobacterial strains**.**

|  | Fold Change | log2(FC) |
| --- | --- | --- |
| Root dry weight | 2.7143 | 1.4406 |
| No. of leaves | 2.2459 | 1.1673 |
| Shoot dry weight | 2.1743 | 1.1206 |
| Root fresh weight | 1.9872 | 0.99072 |
| Shoot Fresh weight | 1.5976 | 0.67591 |
| Shoot height | 1.5803 | 0.66021 |
| Root length | 1.5738 | 0.65425 |

**Tea clone TV1**

1. **Treatment 1 (Strain SA1)**


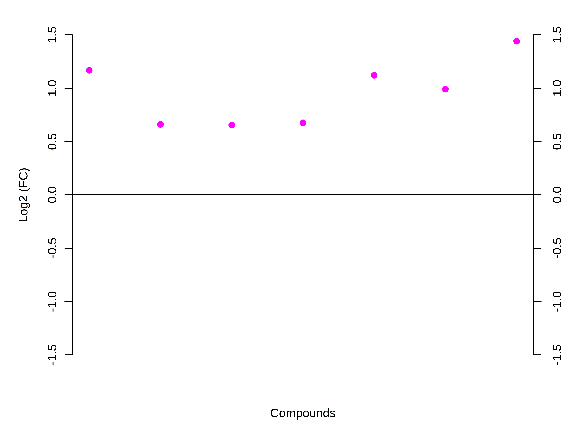


1. **Treatment 2 (Strain T1LA3)**

|  | Fold Change | log2(FC) |
| --- | --- | --- |
| Root dry weight | 6.2198 | 2.6369 |
| Root fresh weight | 3.3504 | 1.7443 |
| Shoot dry weight | 2.6514 | 1.4067 |
| No. of leaves | 2.3443 | 1.2291 |
| Shoot Fresh weight | 1.9538 | 0.96626 |
| Shoot height | 1.8523 | 0.88932 |
| Root length | 1.3393 | 0.42149 |


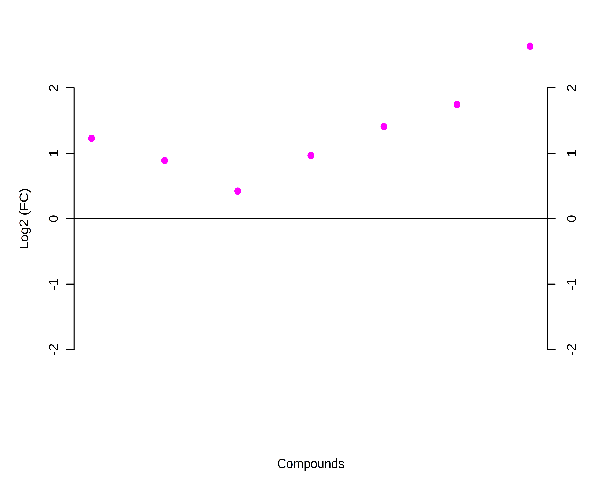


1. **Treatment 3 (Strain SA14)**

|  | Fold Change | log2(FC) |
| --- | --- | --- |
| Root dry weight | 3.4505 | 1.7868 |
| Root fresh weight | 1.9145 | 0.93699 |
| Shoot dry weight | 1.8991 | 0.9253 |
| No. of leaves | 1.6393 | 0.71312 |
| Shoot height | 1.5772 | 0.65739 |
| Shoot Fresh weight | 1.4777 | 0.56339 |
| Root length | 1.3255 | 0.40656 |


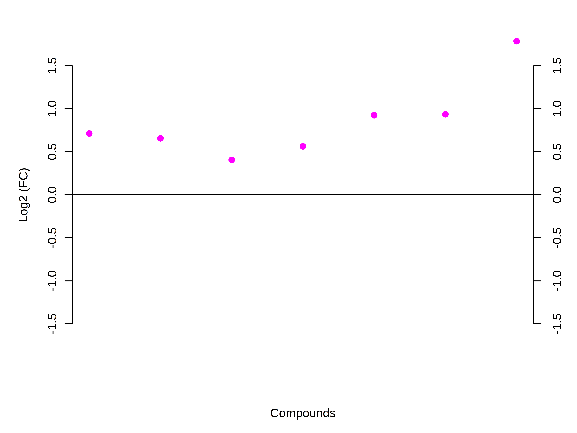


1. **Treatment 4 (SA1+T1LA3+SA14)**

|  | Fold Change | log2(FC) |
| --- | --- | --- |
| Root dry weight | 4.0549 | 2.0197 |
| Root fresh weight | 2.5513 | 1.3512 |
| Shoot dry weight | 1.7339 | 0.79406 |
| No. of leaves | 1.5902 | 0.66918 |
| Shoot Fresh weight | 1.4914 | 0.5767 |
| Root length | 1.4552 | 0.54119 |
| Shoot height | 1.1662 | 0.22176 |


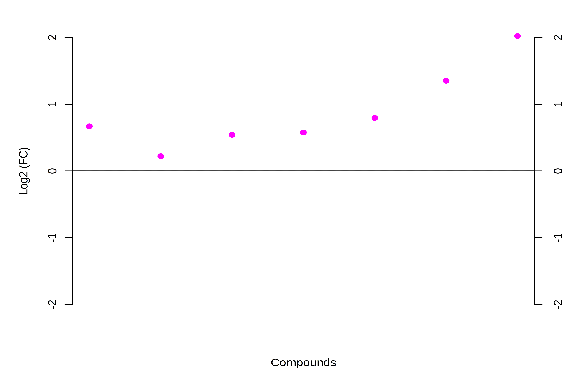


**Tea clone TV9**

**(e)Treatment 1 (Strain SA1)**

|  | Fold Change | log2(FC) |
| --- | --- | --- |
| Root length | 1.7653 | 0.81991 |
| Root dry weight | 1.7121 | 0.77578 |
| No. of leaves | 1.64 | 0.7137 |
| Shoot dry weight | 1.5755 | 0.65578 |
| Shoot height | 1.4196 | 0.50553 |
| Shoot Fresh weight | 1.2558 | 0.32857 |
| Root fresh weight | 1.2135 | 0.27922 |


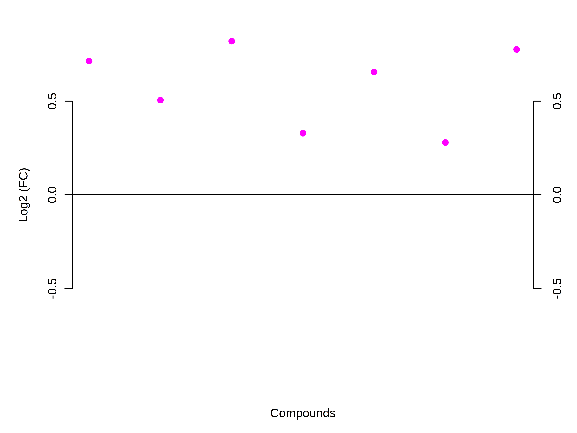


**(f)Treatment 2 (Strain T1LA3)**

|  | Fold Change | log2(FC) |
| --- | --- | --- |
| Root dry weight | 2.5303 | 1.3393 |
| Root length | 1.9026 | 0.92799 |
| No. of leaves | 1.72 | 0.78241 |
| Root fresh weight | 1.4948 | 0.57994 |
| Shoot dry weight | 1.2925 | 0.37011 |
| Shoot height | 1.2226 | 0.28995 |
| Shoot Fresh weight | 1.13 | 0.1763 |


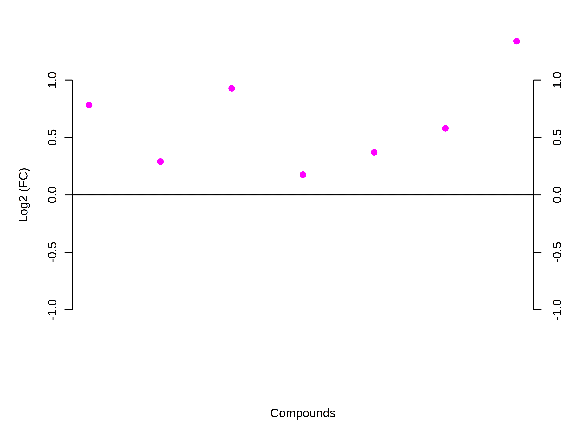


**(g) Treatment 3 (Strain SA14)**

|  | Fold Change | log2(FC) |
| --- | --- | --- |
| Root length | 1.6292 | 0.70418 |
| No. of leaves | 1.48 | 0.5656 |
| Root dry weight | 1.2803 | 0.35649 |
| Shoot height | 1.1499 | 0.20153 |
| Root fresh weight | 1.0651 | 0.090995 |


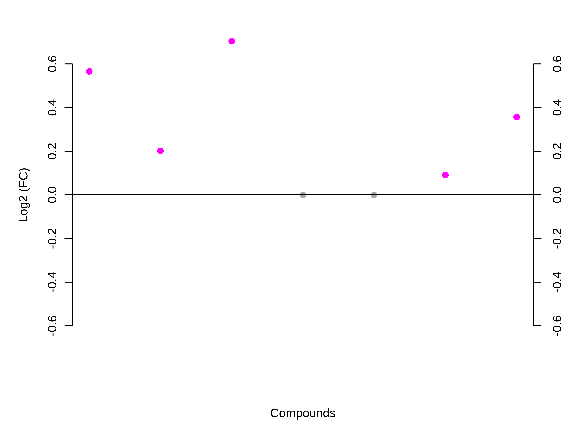


**(h) Treatment 4 (SA1+T1LA3+SA14)**

|  | Fold Change | log2(FC) |
| --- | --- | --- |
| Root length | 1.7653 | 0.81991 |
| Root dry weight | 1.7121 | 0.77578 |
| No. of leaves | 1.64 | 0.7137 |
| Shoot dry weight | 1.5755 | 0.65578 |
| Shoot height | 1.4196 | 0.50553 |
| Shoot Fresh weight | 1.2558 | 0.32857 |
| Root fresh weight | 1.2135 | 0.27922 |


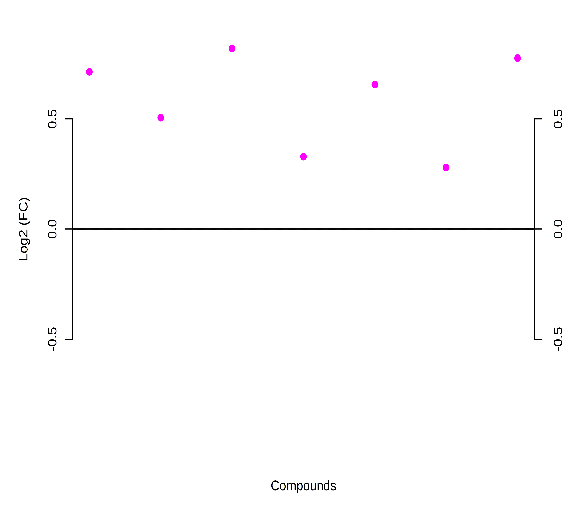


**Tea clone TV 18**

**(i) Treatment 1 (Strain SA1)**

|  | Fold Change | log2(FC) |
| --- | --- | --- |
| Root dry weight | 1.6305 | 0.70532 |
| Root length | 1.6128 | 0.68952 |
| No. of leaves | 1.5946 | 0.67319 |
| Shoot dry weight | 1.5171 | 0.60133 |
| Shoot height | 1.4645 | 0.55041 |
| Root fresh weight | 1.3728 | 0.45708 |
| Shoot Fresh weight | 1.3528 | 0.43594 |


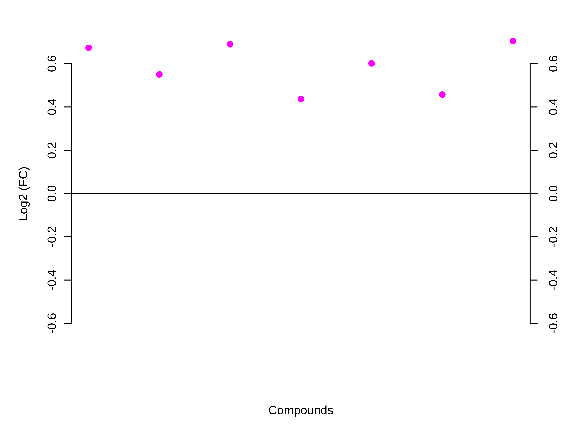


**(j) Treatment 2 (Strain T1LA3)**

|  | Fold Change | log2(FC) |
| --- | --- | --- |
| Root dry weight | 2.0373 | 1.0267 |
| Root fresh weight | 1.5878 | 0.66704 |
| Root length | 1.5348 | 0.61808 |
| Shoot height | 1.5015 | 0.58645 |
| Shoot dry weight | 1.4639 | 0.5498 |
| Shoot Fresh weight | 1.3165 | 0.39668 |
| No. of leaves | 1.1532 | 0.20558 |


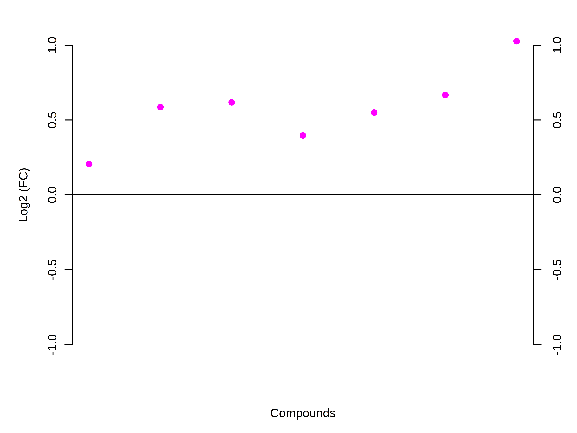


|  | Fold Change | log2(FC) |
| --- | --- | --- |
| Shoot dry weight | 1.8384 | 0.87845 |
| Root length | 1.6517 | 0.72396 |
| Root dry weight | 1.6136 | 0.69025 |
| Shoot Fresh weight | 1.572 | 0.65259 |
| Shoot height | 1.5401 | 0.62305 |
| No. of leaves | 1.3964 | 0.48171 |
| Root fresh weight | 1.3638 | 0.44763 |

**(k) Treatment 3 (Strain SA14)**


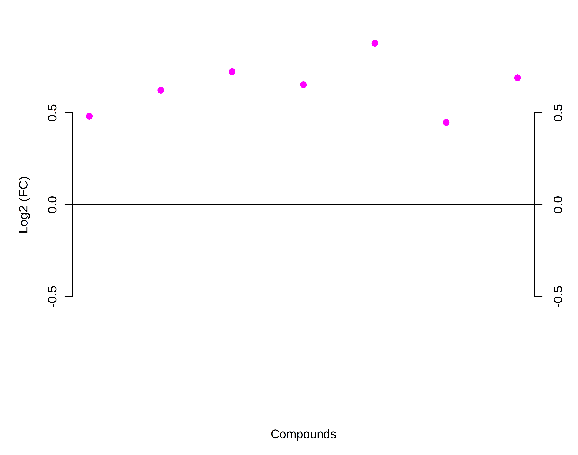


**(l) Treatment 4 (SA1+T1LA3+SA14)**

|  | Fold Change | log2(FC) |
| --- | --- | --- |
| Root dry weight | 1.7695 | 0.82333 |
| Root length | 1.7591 | 0.81488 |
| Shoot height | 1.4666 | 0.55244 |
| Root fresh weight | 1.4462 | 0.5323 |
| No. of leaves | 1.4414 | 0.52751 |
| Shoot dry weight | 1.3897 | 0.47481 |
| Shoot Fresh weight | 1.1271 | 0.17263 |


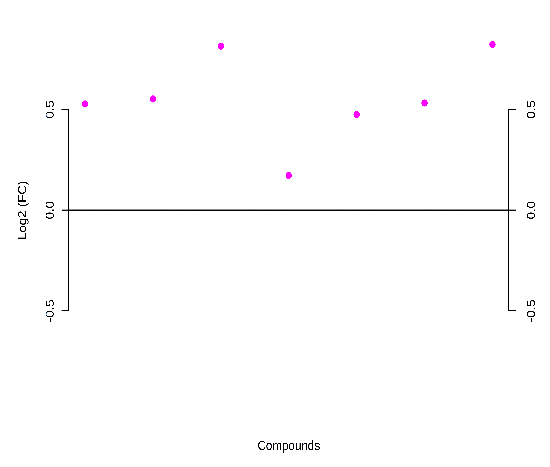


**Tea clone TV 22**

**(m) Treatment 1 (Strain SA1)**

|  | Fold Change | log2(FC) |
| --- | --- | --- |
| No. of leaves | 1.5789 | 0.65896 |
| Root dry weight | 1.4429 | 0.52893 |
| Root length | 1.2918 | 0.36935 |
| Shoot dry weight | 1.2888 | 0.36601 |
| Shoot height | 1.2777 | 0.35357 |
| Root fresh weight | 1.265 | 0.33909 |
| Shoot Fresh weight | 1.2134 | 0.27905 |


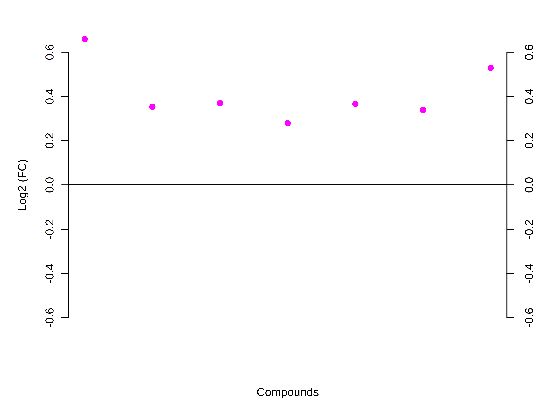


**(n) Treatment 2 (Strain T1LA3)**

|  | Fold Change | log2(FC) |
| --- | --- | --- |
| Root dry weight | 1.6629 | 0.73366 |
| Root length | 1.4964 | 0.58147 |
| Root fresh weight | 1.3966 | 0.4819 |
| No. of leaves | 1.3947 | 0.47999 |
| Shoot height | 1.2856 | 0.36248 |
| Shoot dry weight | 1.1813 | 0.24033 |
| Shoot Fresh weight | 1.1339 | 0.18134 |


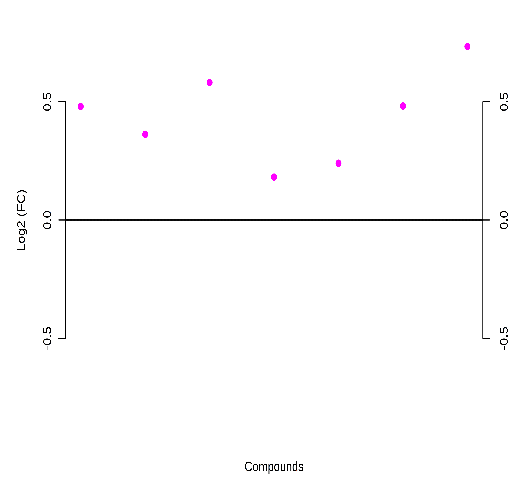


**(o) Treatment 3 (Strain SA14)**

|  | Fold Change | log2(FC) |
| --- | --- | --- |
| Root length | 1.931 | 0.94934 |
| Root dry weight | 1.5971 | 0.67549 |
| Root fresh weight | 1.3573 | 0.4407 |
| Shoot dry weight | 1.3149 | 0.39495 |
| No. of leaves | 1.2895 | 0.36678 |
| Shoot Fresh weight | 1.2327 | 0.30181 |
| Shoot height | 1.0837 | 0.11598 |


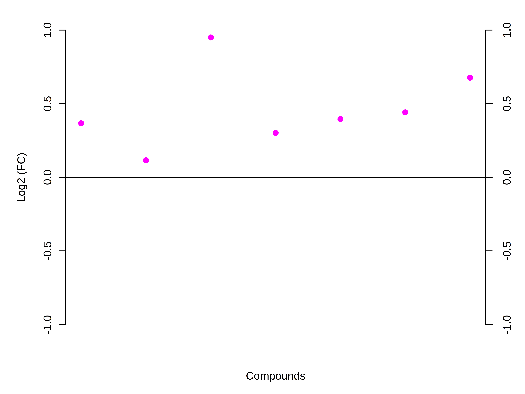


**(p) Treatment 4 (SA1+T1LA3+SA14)**

|  | Fold Change | log2(FC) |
| --- | --- | --- |
| Root dry weight | 2.0343 | 1.0245 |
| Root fresh weight | 1.6188 | 0.69493 |
| No. of leaves | 1.3947 | 0.47999 |
| Root length | 1.339 | 0.42114 |
| Shoot height | 1.237 | 0.30683 |
| Shoot dry weight | 1.169 | 0.22524 |
| Shoot Fresh weight | 1.1249 | 0.16974 |


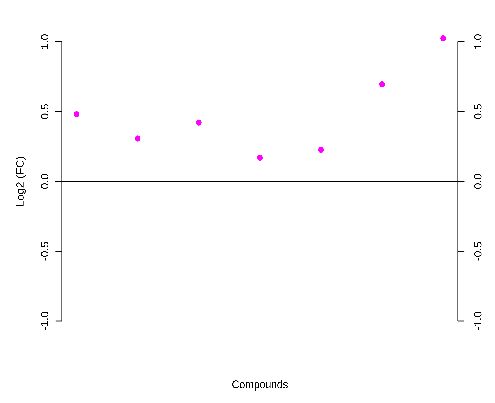


Note: In all figures the red dots represent parameters above the threshold and Important features selected by fold change analysis with threshold 1. The values are on log scale, so that both up-regulated and down regulated parameters can be plotted in a symmetrical way

**Supplementary table 1|** Solubilization of inorganic phosphate in liquid Pikovskaya’s medium with different initial values of pH for endophytic actinobacteria isolates obtained from *Camellia* spp., after 6 days of incubation at 28 °C

| **Sl. No.** | **Isolate code** | **pH 6.0** | **pH 7.0** | **pH 8.0** |
| --- | --- | --- | --- | --- |
| 1 | SA1 | 47.0 ± 6.0 | 104.6 ± 10.7 | 49.0 ± 11.0 |
| 2 | SA14 | 149.1 ± 9.4 | 223.3 ± 14.3 | 86.8 ± 7.3 |
| 3 | S85 | 118.3 ± 17.3 | 164.0 ± 14.0 | 92.6 ± 8.4 |

**Supplementary table 2|** Soil chemical parameters

1. **Tea clone TV1**

| **Soil Parameters** | **Control** | **SA1** | **T1LA3** | **SA14** | **SA1+T1LA3+SA14** |
| --- | --- | --- | --- | --- | --- |
| Potassium (as K), % by mass | 0.032 | 0.027 | 0.021 | 0.033 | 0.029 |
| Organic Matter, % by mass | 0.55 | 0.46 | 0.46 | 0.55 | 0.55 |
| Total Phosphorus (as P), % by mass | 0.065 | 0.19 | 0.017 | 0.029 | 0.029 |
| Available Phosphorus (as P), % by mass | 0.021 | 0.026 | 0.009 | 0.007 | 0.007 |
| Total Nitrogen (as N), % by mass | 0.08 | 0.028 | 0.1 | 0.09 | 0.09 |
| Available Nitrogen (as N), % by mass | 0.011 | 0.01 | 0.02 | 0.016 | 0.014 |
| Sulphur (as So₄), % by mass | 0.096 | 0.15 | 0.073 | 0.6 | 0.21 |
| Calcium (as Ca) * % by mass | ND (DL=0.001) | ND (DL=0.001) | ND (DL=0.001) | ND (DL=0.001) | ND (DL=0.001) |
| Magnesium (as Mg) *, % by mass | 0.14 | 0.3 | 0.11 | 0.12 | 0.1 |
| Water Holding Capacity*, % by mass | 48.18 | 39.75 | 40.33 | 45.9 | 38.33 |
| Manganese (as Mn) *, mg/kg | 159.28 | 141.97 | 163.59 | 149.57 | 140.5 |
| Iron (as Fe) *, mg/kg | 64.6 | 61.15 | 72.45 | 69.07 | 64.29 |
| Copper (as Cu) *, mg/kg | 2.57 | 2.92 | 2.87 | 2.96 | 2.69 |
| Zinc (as Zn) *, mg/kg | 16.04 | 17.89 | 19.12 | 16.28 | 16.41 |
| Molybdenum (as Mo) *, mg/kg | 1.23 | ND (DL=0.25) | ND (DL=0.25) | ND (DL=0.25) | ND (DL=0.25) |
| Boron (as B) *, mg/kg | 1.59 | 12.86 | 1.23 | 1.15 | 1.13 |

1. **Tea clone TV9**

| **Soil parameters** | **Control** | **SA1** | **T1LA3** | **SA14** | **SA1+T1LA3+SA14** |
| --- | --- | --- | --- | --- | --- |
| Potassium (as K), % by mass | 0.044 | 0.044 | 0.047 | 0.051 | 0.055 |
| Organic Matter, % by mass | 0.91 | 0.55 | 0.68 | 0.9 | 0.92 |
| Total Phosphorus (as P), % by mass | 0.031 | 0.12 | 0.037 | 0.044 | 0.039 |
| Available Phosphorus (as P), % by mass | 0.011 | 0.036 | 0.008 | 0.006 | 0.009 |
| Total Nitrogen (as N), % by mass | 0.16 | 0.05 | 0.17 | 0.15 | 0.16 |
| Available Nitrogen (as N), % by mass | 0.036 | ND(DL=0.001) | 0.022 | 0.062 | 0.071 |
| Sulphur (as So₄), % by mass | 0.18 | 0.21 | 0.29 | 0.48 | 0.31 |
| Calcium (as Ca)* % by mass | ND (DL=0.001) | ND (DL=0.001) | ND (DL=0.001) | ND (DL=0.001) | ND (DL=0.001) |
| Magnesium (as Mg)*, % by mass | 0.13 | 0.13 | 0.14 | 0.13 | 0.12 |
| Water Holding Capacity*, % by mass | 42.18 | 39.32 | 48.18 | 45.55 | 46.3 |
| Manganese (as Mn)*, mg/kg | 97.01 | 119.36 | 4.61 | 6.16 | 10.16 |
| Iron (as Fe)*, mg/kg | 64.68 | 61.34 | 2.38 | 3.2 | 7.11 |
| Copper (as Cu)*, mg/kg | 3.29 | 2.65 | ND(DL=1.0) | ND(DL=1.0) | ND(DL=1.0) |
| Zinc (as Zn)*, mg/kg | 19.04 | 16.52 | ND(DL=1.0) | ND (DL=1.0) | ND (DL=1.0) |
| Molybdenum (as Mo)*, mg/kg | ND (DL=0.25) | ND (DL=0.25) | ND (DL=0.25) | ND (DL=0.25) | ND (DL=0.25) |
| Boron (as B)*, mg/kg | 1.86 | ND(DL=1.0) | ND(DL=1.0) | ND (DL=0.25) | ND (DL=0.25) |

1. **Tea clone TV18**

| **Soil parameters** | **Control** | **SA1** | **T1LA3** | **SA14** | **SA1+T1LA3+SA14** |
| --- | --- | --- | --- | --- | --- |
| Potassium (as K), % by mass | 0.017 | 0.021 | 0.02 | 0.02 | 0.031 |
| Organic Matter, % by mass | 0.46 | 0.69 | 0.42 | 0.43 | 0.42 |
| Total Phosphorus (as P), % by mass | 0.017 | 0.03 | 0.14 | 0.16 | 0.12 |
| Available Phosphorus (as P), % by mass | 0.008 | ND(DL=0.001) | 0.03 | 0.029 | 0.037 |
| Total Nitrogen (as N), % by mass | 0.11 | 0.14 | 0.15 | 0.15 | 0.11 |
| Available Nitrogen (as N), % by mass | 0.036 | 0.042 | 0.05 | 0.06 | 0.061 |
| Sulphur (as So₄), % by mass | 0.1 | 0.065 | 0.091 | 0.096 | 0.13 |
| Calcium (as Ca)* % by mass | ND (DL=0.001) | ND (DL=0.001) | ND (DL=0.001) | ND (DL=0.001) | ND (DL=0.001) |
| Magnesium (as Mg)*, % by mass | 0.14 | 0.14 | 0.11 | 0.13 | 0.12 |
| Water Holding Capacity*, % by mass | 44.52 | 44.49 | 41.92 | 43.97 | 45.32 |
| Manganese (as Mn)*, mg/kg | 4 | 6.47 | ND (DL=1.0) | ND (DL=1.0) | ND (DL=1.0) |
| Iron (as Fe)*, mg/kg | ND(DL=5.0) | ND(DL=5.0) | ND(DL=5.0) | ND(DL=5.0) | ND(DL=5.0) |
| Copper (as Cu)*, mg/kg | ND(DL=1.0) | ND(DL=1.0) | ND(DL=1.0) | ND(DL=1.0) | ND(DL=1.0) |
| Zinc (as Zn)*, mg/kg | ND(DL=1.0) | ND(DL=1.0) | ND(DL=1.0) | ND(DL=1.0) | ND(DL=1.0) |
| Molybdenum (as Mo)*, mg/kg | ND (DL=0.25) | ND (DL=0.25) | ND (DL=0.25) | ND (DL=0.25) | ND (DL=0.25) |
| Boron (as B)*, mg/kg | ND(DL=1.0) | ND(DL=1.0) | ND(DL=1.0) | ND(DL=1.0) | ND(DL=1.0) |

1. **Tea clone TV22**

| Soil parameters | **Control** | **SA1** | **T1LA3** | **SA14** | **SA1+T1LA3+SA14** |
| --- | --- | --- | --- | --- | --- |
| Potassium (as K), % by mass | 0.04 | 0.02 | 0.022 | 0.02 | 0.043 |
| Organic Matter, % by mass | 0.69 | 0.51 | 0.63 | 0.61 | 0.74 |
| Total Phosphorus (as P), % by mass | 0.16 | 0.18 | 0.038 | 0.097 | 0.03 |
| Available Phosphorus (as P), % by mass | 0.024 | 0.029 | 0.009 | 0.03 | 0.007 |
| Total Nitrogen (as N), % by mass | 0.16 | 0.15 | 0.13 | 0.12 | 0.17 |
| Available Nitrogen (as N), % by mass | 0.07 | 0.08 | 0.07 | 0.08 | 0.06 |
| Sulphur (as So₄), % by mass | 0.085 | 0.068 | 0.22 | 0.076 | 0.18 |
| Calcium (as Ca)* % by mass | ND (DL=0.001) | ND (DL=0.001) | ND (DL=0.001) | ND (DL=0.001) | ND (DL=0.001) |
| Magnesium (as Mg)*, % by mass | 0.11 | 0.13 | 0.03 | 0.14 | 0.14 |
| Water Holding Capacity*, % by mass | 42.41 | 42.43 | 39.95 | 42.79 | 43.76 |
| Manganese (as Mn)*, mg/kg | ND (DL=1.0) | 3.11 | 3.71 | 1.5 | 1.56 |
| Iron (as Fe)*, mg/kg | ND(DL=5.0) | ND(DL=5.0) | ND(DL=5.0) | ND(DL=5.0) | ND(DL=5.0) |
| Copper (as Cu)*, mg/kg | ND(DL=1.0) | ND(DL=1.0) | ND(DL=1.0) | ND(DL=1.0) | ND(DL=1.0) |
| Zinc (as Zn)*, mg/kg | ND(DL=1.0) | ND(DL=1.0) | ND(DL=1.0) | ND(DL=1.0) | ND(DL=1.0) |
| Molybdenum (as Mo)*, mg/kg | ND (DL=0.25) | ND (DL=0.25) | ND (DL=0.25) | ND (DL=0.25) | ND (DL=0.25) |
| Boron (as B)*, mg/kg | ND(DL=1.0) | ND(DL=1.0) | ND(DL=1.0) | ND(DL=1.0) | ND(DL=1.0) |
